# The leaf beetle *Labidostomis lusitanica* (Coleoptera: Chrysomelidae) as an Iberian pistachio pest: projecting risky areas

**DOI:** 10.1002/ps.6624

**Published:** 2021-09-16

**Authors:** Sara Rodrigo Gómez, Diego Gil‐Tapetado, Jaime García‐Gila, Javier Blasco‐Aróstegui, Carlo Polidori

**Affiliations:** ^1^ Instituto de Ciencias Ambientales (ICAM) Universidad de Castilla‐La Mancha Toledo Spain; ^2^ Instituto Regional de Investigación y Desarrollo Agroalimentario y Forestal (IRIAF)‐Centro de Investigación Agroambiental “El Chaparrillo” Ciudad Real Spain; ^3^ Departamento de Biodiversidad, Ecología y Evolución Universidad Complutense de Madrid Madrid Spain; ^4^ Museo Nacional de Ciencias Naturales (MNCN‐CSIC) Madrid Spain; ^5^ CIBIO‐InBIO, Centro de Investigação em Biodiversidade e Recursos Genéticos da Universidade do Porto Vairão Portugal; ^6^ Dipartimento di Scienze e Politiche Ambientali Università degli Studi di Milano Milan Italy

**Keywords:** leaf beetle, chrysomelidae, potential distribution model, phenology, survey

## Abstract

**BACKGROUND:**

Pistachio (*Pistacia vera* L.) is a commercially important tree in the Mediterranean basin, where there is a considerable increase in cultivation, especially in Spain. Because of its recent introduction as a crop in the country (1980s), studies on the pests of pistachio in Spain are still rare. Here, we studied the leaf beetle *Labidostomis lusitanica* (Coleoptera: Chrysomelidae), which was observed on pistachio and might become a serious pest under the expanding Spanish pistachio fields. Because early detection of pests is extremely important to properly plan control strategies, we (i) updated the information on the distribution of the species through samplings and surveys, and (ii) modelled its potential distribution.

**RESULTS:**

Currently, *L. lusitanica* occurs across the whole Iberian Peninsula, especially in its southern and eastern parts, with adults on flight roughly from late April to early June. Analysis of climatic niches showed that *L. lusitanica* prefers dry and hot areas, which are conditions found especially in the central‐southern parts of the Iberian Peninsula. Such highly suitable areas for this pest overlap considerably with the suitable areas for pistachio cultivation. Surveys of pistachio growers weakly suggested a higher pest attack probability, but, unexpectedly, a lower perceived impact in very suitable areas for *L. lusitanica,* suggesting that other factors may shape its pest potential in a complex way.

**CONCLUSION:**

In line with what has been observed for other *Labidostomis* species on pistachio in other Mediterranean countries, *L. lusitanica* has a good potential to harm pistachio production in Spain, claiming for further investigations and prevention strategies. © 2021 The Authors. *Pest Management Science* published by John Wiley & Sons Ltd on behalf of Society of Chemical Industry.

## Graphical abstract

The beetle *Labidostomis lusitanica* occurs mostly in the central‐southern and eastern part of the Iberian Peninsula, in dry and hot climates. Models predict a considerable overlap between the most suitable areas for the beetle and those for pistachio cultivation, suggesting a high pest potential.

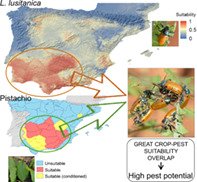

## 1 INTRODUCTION

Pistachio (*Pistacia vera* L.) (Anacardiaceae) is a dioecious and deciduous tree, native to western Asia and Asia Minor and also cultivated in Europe for about 2000 years, especially in the Mediterranean basin, to produce edible nuts.^1^ Pistachio trees are ≤12 m high, with compound‐pinnate, ovoid‐shaped leaves and apetalous flowers which are borne in panicles; pollen is spread by wind and the flowering period covers up to two weeks during the spring.^2^, ^3^ In the Mediterranean basin, pistachio is a commercially relevant crop in Greece, Italy and Spain.^4^ In Spain, since its introduction in the 1980s, it has seen an increase in cultivation coverage, especially in recent years, from just over 20 000 ha in 2017^5^, ^6^ to ≈40 000 ha according to the latest available data.^7^ Currently, at the European level, Spain has the greatest pistachio crop area since 2014, approximately six‐fold greater than Greece and about seven‐fold greater than Italy. Within Spain, Castilla‐La Mancha is the autonomous community that brings together most of this area (≈75% of the national total).^8^ However, Spanish production has not yet exceeded that of Greece and Italy.^4^

Owing to the recent entry as a crop in the country and the even more recent great expansion, studies on the pests of pistachio in Spain are still lacking, and the sparse, often anecdotical available information suggests that the Spanish pistachio has a low incidence of pests, compared to other countries which traditionally cultivate it.^8^, ^9^, ^10^ However, it is foreseeable that a huge growth in pistachio cultivation in Spain may lead to a progressively higher occurrence and abundance of new pests or to an expansion of existing ones. For example, in California's pistachio areas that were once considered ‘essentially pest‐free’ a number of important pests are now recorded.^11^ For this reason, it is important to monitor in detail the pistachio areas in the Iberian Peninsula, and to study the biology and ecology of their newly established pests.

One of the insect groups with an emergent potential to become a serious problem for Pistachio in Spain is the leaf beetle tribe Clytrini (Coleoptera: Chrysomelidae), which comprises ≈50 species in the Iberian Peninsula.^12^ Adults of Clytrini beetles are polyphagous and feed largely on flowers, buds, young leaves and pollen,^13^, ^14^ whereas larvae are detritivorous and myrmecophiles, and are found on the soil, commonly near ant nests.^15^, ^16^, ^17^

Among Clytrini, *Labidostomis lusitanica* (Germar, 1824) is a Western Mediterranean polyphagous species^18^ which in Spain has been recorded as a pest of the grapevine (*Vitis vinifera* L., Vitaceae), plum tree (*Prunus* sp., Rosaceae)^19^ and occasionally avocado (*Persea americana* Mill., Lauraceae).^20^ There also are circumstantial reports of *L. lusitanica* feeding on pistachio leaves,^5^ although its possible threat as a pest for this crop is still not clear. Hence, a deeper picture on its potential as a pest of this crop is important. The ‘Guide for pistachio pest management’ of the Ministerio de Agricultura y Pesca, Alimentación y Medio Ambiente of Spain (MAPAMA, today changed to MAPA) reported *L. lusitanica* as a voracious pistachio leaf feeder that can completely defoliate young trees in few hours.^5^ Two congeneric species, *Labidostomis diversifrons* Lefevre, 1876 and *Labidostomis longimana* (Linnaeus, 1758) have been recorded on pistachio trees in Syria and Turkey, respectively, causing important injuries to the leaves which make the trees weaker.^21^, ^22^ Hence, *L. lusitanica* might become a serious pest of extensively cultivated *P. vera*, and the currently rapid increasing surface of this crop in central and southern Spain could be affected by this leaf beetle in the future.

In this study, we aimed to provide new information on *L. lusitanica* in Spanish pistachio fields. In particular, we (i) updated the information on Spanish distribution of the species in pistachio fields through sampling (in Castilla‐La Mancha) and surveys completed by pistachio growers, and (ii) modelled the potential distribution of the species and compared it with the suitable areas for pistachio cultivation at the Iberian Peninsula level.

## 2 MATERIALS AND METHODS

### 2.1 *L. lusitanica* sampling

**Figure 1:**
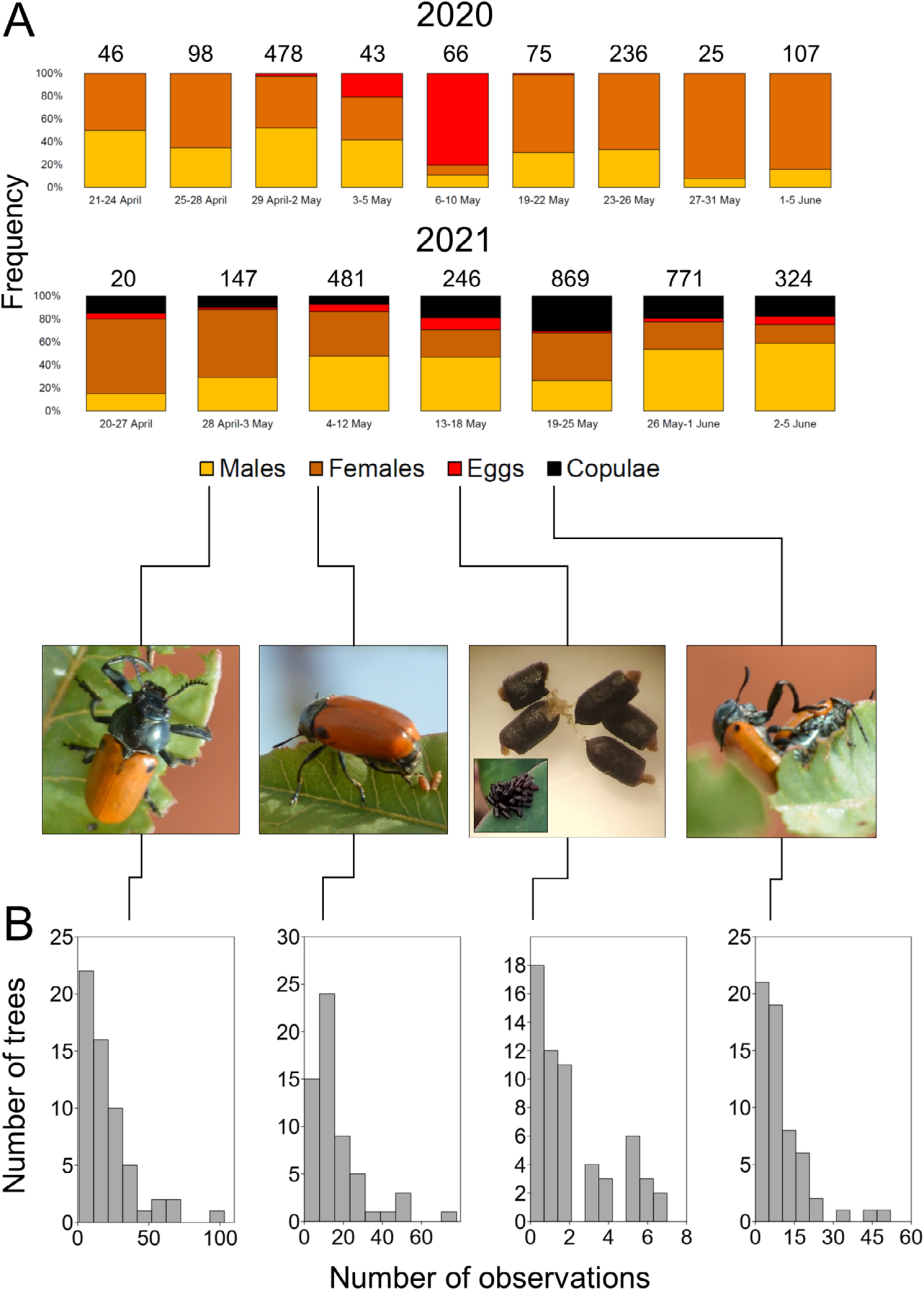
(A) Frequency of collected/observed individuals (males, females, egg clusters and mating pairs shown in pictures) across the study period (above, 2020; below, 2021). Numbers above the bars indicate the sample size. (B) Number of trees where different number of (left to right) males, females, egg clusters and mating pairs have been observed across the 59 pistachio trees in 2021.

Collections of beetles were carried out in 2020 and 2021 in pistachio plantations located in Castilla‐La Mancha, in the following municipalities: Torrenueva, Calzada de Calatrava, Pozuelo de Calatrava, Torralba de Calatrava, Fernán Caballero, Miguelturra, El Chaparrillo, Porzuna, Villarrubia de los Ojos, Manzanares and Ciudad Real (province of Ciudad Real); Madridejos, Quintanar de la Orden, Calera y Chozas and Villatobas (province of Toledo); Mondéjar (province of Guadalajara); Minaya (province of Albacete); Villamayor de Santiago and Cuenca (province of Cuenca) (Supporting Information, Table S1). Locations cover all four provinces of Castilla‐La Mancha. Additionally, sampling was carried out at two localities outside Castilla‐La Mancha: Vilches (province Jaén, region Andalusia) and Badajoz (province Badajoz, region Extremadura). Transects with annotation of direct observations and/or with capture by entomological nets^23^ were used to record the species. Direct records by observation were possible because of the easily distinguishable morphology of this species, with adults (6–12 mm long) having a dark metallic thorax and orange elytra with two black dots, one on each elytron [Fig. 1(A)]. Furthermore, sexes can be distinguished easily, because males have longer front legs than females and a thorax that also bigger than that of females [Fig. 1(A)]. The eggs, brownish, cylindrical and conical at their vertex, are laid in clusters of about ten on the host plant leaves, and thus also are easily associable to this species during observations [Fig. 1(A)]. All of the collected specimens were placed in tubes and frozen or preserved in 70% ethanol to later confirm species and sex identification.

Adults were sampled both directly (transects) and indirectly (traps), and egg clusters were recorded on plants only during transects. Collections were carried out in 2020 from 24 April to 10 May and from 19 May to 5 June, and in 2021 from 20 April to 5 June, in both years every four to five days. This resulted in a total of nine sampling periods in 2020 and seven sampling periods in 2021. Owing to logistic reasons, in 2020 not all sites were sampled in all periods. Hence, the 2020 collected data provide useful information on occurrence and general abundance of *L. lusitanica* in the region and overall rough phenology, but cannot be used for intersite comparisons. In 2021, sampling was possible for the whole period in one single locality (El Chaparrillo), thus allowing a better inspection of phenology while avoiding possible confounding interlocality effects. At that site in 2021 sampling also was more properly structured. In particular, we sampled beetles on 59 individually marked pistachio trees during the whole study. Sampling on the individual trees was set at random rotation, and each tree received at the end of the study a total of seven visits, that is one for each of the seven sampling periods during the study. At this site in 2021, males and females in copula were also recorded.

We tested for a clumped distribution of males, females, copulae and egg clusters across trees (i.e. few trees accumulating most of the observations) using the Shapiro–Wilk test for normality and calculating the skewness of the distributions. Normality would suggest that observations are likely to occur with similar frequencies on all trees, whereas high skewness values would indicate either a higher concentrations of observations on few (left‐skewed) or many (right‐skewed) trees. To test if trees with more males observed also were those with more females and copulae observed, we performed a Pearson correlation test. The same test was used to verify associations between the number of copulae and the number of egg clusters, and between the number of copulae and the number of egg cluster relative to the number of copulae, across trees.

### 2.2 Farmer survey

Free software provided by Google (google forms) was used as a tool to develop and disseminate a questionnaire to pistachio farmers across 15 Spanish provinces. On the whole, 253 farmers were contacted for the survey. The survey was carried out from March to June 2020. The questionnaire was structured in two main blocks: (i) general data [municipality, surface of the plantation (ha), % of the plantation of 0–3, 4–8 and >8 years of age), (ii) data on *L. lusitanica* [occurrence (binary: yes/no) and perceived tree loss due to the beetle (0 = no loss, 1 = <10%, 2 = ≥10%). Pictures were provided to the farmers in order to help their identification of the beetles and damage on the pistachio leaves. To verify if attack probability (yes/no occurrence answer) depended on plantation area, % of plantation of different ages and *L. lusitanica* suitability (obtained as detailed below) (continuous explanatory variables), we used binary logistic regressions. Hence, the binary outcome was assessed with numbers of positive and negative responses to the beetle detection (i.e. attack probability). If the proportion of positive responses was statistically significantly larger than negative responses, then the attack probability increased with increasing values of the independent variable. To verify if the impact (ranks of perceived loss) depended on the same explanatory variables, we used ordered logistic regressions, which are adequate in case of ranked values of the response variable. In both cases, one regression was done *per* independent variable. These statistics were performed in R v3.5.0,^24^ through rstudio Software v1.1.453.^25^

### 2.3 Actual distributions of *L. lusitanica*

We described the current distribution of *L. lusitanica* in the Iberian Peninsula using available records in published articles, GBIF (Global Biodiversity Information Facility),^26^ and the webpages Biodiversidad Virtual^27^ and iNaturalist^28^ (only confirmed identifications of georeferenced photographs were used). Our own new records obtained through the above‐described sampling carried out in 2020 and 2021 then were added (Table S1). In total, 224 georeferenced records were used to map the actual distribution of the species. We kept 207 occurrence points (literature + our 2020 records) to develop all of the analyses on the climatic niche and the potential distribution, whereas the 18 occurrence points recorded through the 2021 sampling were used for *a posteriori* validation of the final model (see below), a method used recently in a similar study.^29^

### 2.4 Climatic niche of *L. lusitanica*

**Figure 2:**
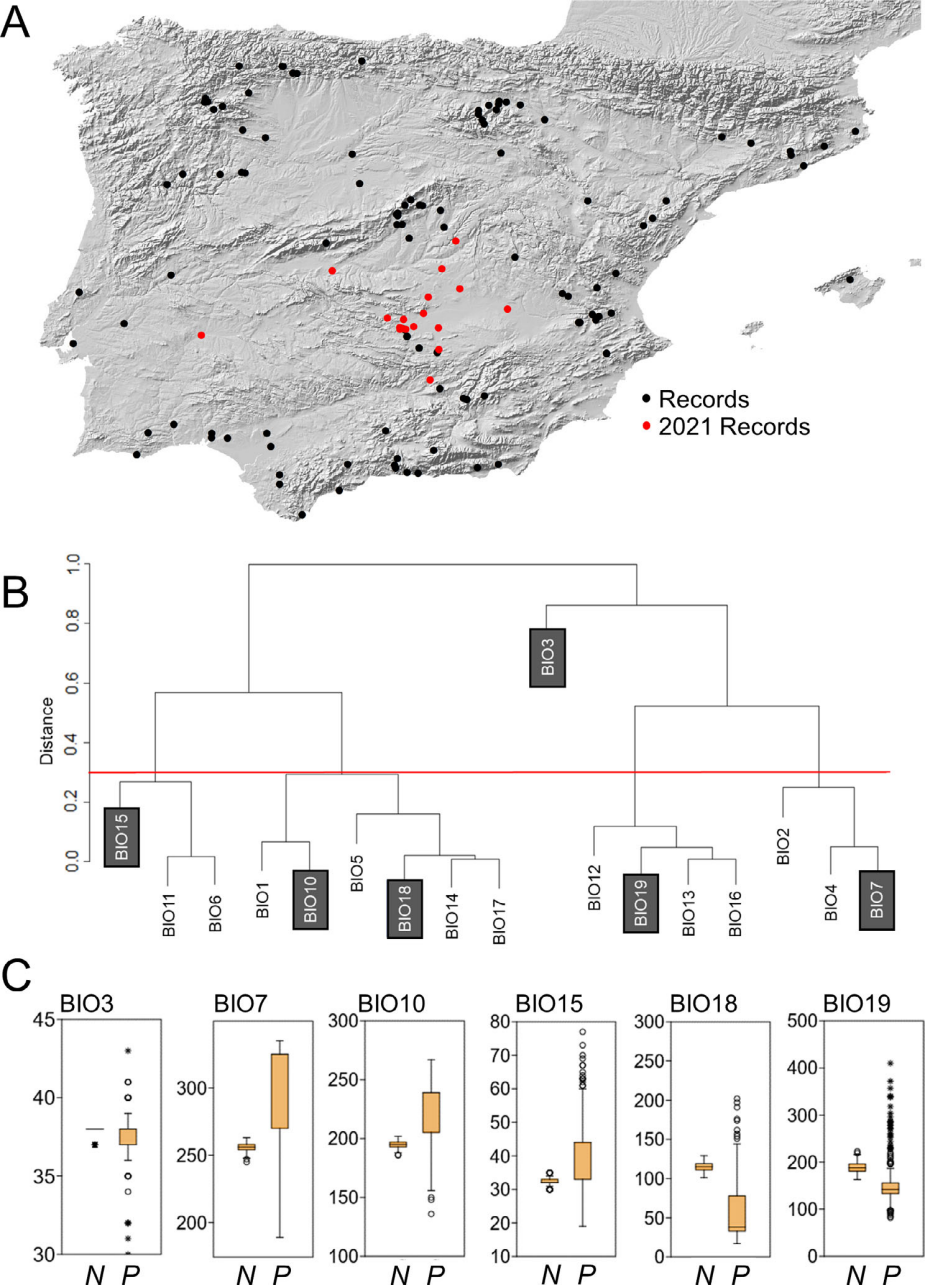
(A) Current distribution of *L. lusitanica* in the Iberian Peninsula, as obtained from this study as well as from previous available information (224 record, see Table S1); red data are the 18 records from 2021 sampling that were used for the *a posteriori* validation of the SDM. (B) Dendrogram obtained through the cluster analysis that was employed to select the relevant BioClim variables (highlighted in grey); the red horizontal line indicates the chosen distance‐threshold to form the clusters (0.3). (C) Differences in the values of the selected BioClim variables between the *L. lusitanica* presence points (*P*) and points with no records (*N*). The latter points were chosen 100 times randomly across the cells with no records and then average values were used. Box‐and‐whisker plots show medians (horizontal lines within boxes), 1st and 3rd quartiles (horizontal lines closing the boxes), and maximum and minimum values (ends of the whiskers). Outliers with a value >1.5 times the interquartile range are shown as circles, whereas values with over three times the interquartile range are shown as stars.

The climatic niche of *L. lusitanica* was calculated using information from 17 layers in the current climate available in WorldClim database v2.1 (http://www.worldclim.org), that is, all layers except BIO8 and BIO9, which represent very biased variables in the Iberian Peninsula.^30^, ^31^ These bioclimatic variables, which have 30 s cell size (1 × 1 km), represent seasonality trends, and average and extreme values of temperatures and precipitation over the period 1970–2000, and are overall ecologically meaningful for species.^32^, ^33^, ^34^, ^35^ Because some climatic variables tend to be strongly intercorrelated, we first performed a hierarchical cluster analysis (using the Ward method) to show the similarities among them^36^ and their partition into subsets (clusters) based on distances.^37^ Among the 17 variables, we then selected only one derived variable from each of the clusters. For each cluster, we selected the most informative variable among those that passed the distance‐threshold of 0.3 (<70% correlation). Thus, for example, if both mean annual temperature and annual range of temperature were included in a particular cluster and passed the distance‐threshold, we chose annual range of temperature as it is more informative. One further variable constituting one exclusive cluster at >0.3 distance was also added because it was not correlated with any of the other variables. A final step was performed to eliminate those variables that overestimated the variance and contributed the most redundant information to the model [variance inflation factor (VIF) > 5].^38^ The final set of selected variables includes BIO3 [Isothermality; i.e. day‐to‐night temperatures oscillations relative to the summer‐to‐winter (annual) oscillations], BIO7 (Temperature annual range), BIO10 (Mean temperature of warmest quarter), BIO15 (Precipitation seasonality; i.e. variability of precipitation during the year), BIO18 (Precipitation of warmest quarter) and BIO19 (Precipitation of coldest quarter) [Fig. 2(B)].

Possible directional preferences of the species for the climatic conditions expressed by the selected variables were evaluated by comparing, with Student's *t*‐tests, the mean values of the variables of the occurrence points with the mean values of the variables of points with no records that fall outside the lower and upper models (see below). The latter were calculated by choosing the same number of occurrence points randomly across the cells in such areas with no records (average of 100 randomly chosen sets of points). These statistics were performed in PAST 3.4.^39^

### 2.5 Potential distributions of *L. lusitanica*

In order to predict the potential distribution of *L. lusitanica* we used the climatic variables selected above in order to build the geographical representation of its climatic niche (i.e. the geographical area in which the climatic environment is suitable for them to live).^40^, ^41^, ^42^, ^43^ We estimated the potential distribution of *L. lusitanica* through the following six species distribution models (SDMs) largely used in biological invasion studies: generalized linear model (GLM),^44^ generalized additive model (GAM),^45^ artificial neural network (ANN),^46^ Classification Tree Analysis (CTA),^47^ maximum entropy (MaxEnt v3.3.0)^48^ and Random Forest (RF).^49^ These six different algorithms were calculated with the *biomod2* library.^50^ The average consensus model based on 100 iterations of these six algorithms was used to predict the potential distribution of the species.^29^

Background and pseudoabsences were constructed through a simple environmental coverage model with only presences, performed with the range between the maximum and minimum values of each selected variable.^51^ Areas habitable to *L. lusitanica* were those areas that had all their values within the maximum and minimum range of each selected variable and such areas were used to establish the background points. However, those areas that did not fulfil at least two of these variables were used to establish the pseudoabsences points.^52^ Presence, pseudoabsence and background data were split 75%/25% to generate an external area (AUC) under the receiver operating characteristic curve (ROC) evaluation for the final models, independently of the internal AUC evaluation of each individual model generated by *biomod2*. A total of 600 individual models were tested with their individual AUC evaluation, only choosing the models with AUC > 0.7 (i.e. good‐to‐excellent performance^53^). All of the models were validated and fulfilled this condition. A final consensus average model and the upper and lower bounds models then were obtained. Finally, the consensus and upper and lower bounds models were evaluated through the external AUC test with the 25% of data. A cut‐off value of 0.55 was used to discriminate overall suitable from unsuitable areas for *L. lusitanica*. R v3.5.0 was used for variables selection, VIF analysis and SDMs. Background and pseudoabsences point generation and model maps were performed in ArcGIS for desktop v10.3.^54^

Finally, to evaluate if the optimal climatic niche – and hence the most suitable potential areas – of *L. lusitanica* overlaps with the best for pistachio cultivation, we digitized and built up a suitability map for pistachio using the climatic variables provided in Couceiro *et al*.,^8^ which used information on climate retrieved from a selected number of meteorological stations across all Spanish provinces. Such data then were compared with the conditions known to positively affect pistachio cultivation, and in case of good conditions, further data regarding soil properties (e.g. texture, permeability) were added to the analyses in order to ultimately define each province as either suitable, unsuitable (i.e. at least one variable have values falling outside the optimal range for pistachio) or suitable with restrictions (i.e. the pistachio should be planted early during spring to assure a successful cultivation). More details of the whole procedure are found in Couceiro *et al*.^8^ We projected all of the information provided in that work on a map of Spain which shows suitable areas, unsuitable areas and areas that are suitable with restrictions with different colours. To test if *L. lusitanica* potential risk is higher in the suitable areas for pistachio, we compared by ANOVA the leaf beetle suitability (as obtained from the SDM average model, see above) across the three suitability categories for pistachio, using 1 × 1 km cells. These statistics were performed in R v3.5.0.

## 3 RESULTS

### 3.1 Activity and current distribution

The samples summed up a total of 1174 (in 2020) and 2286 (in 2021) individuals + egg clusters, with abundances varying greatly across the study periods within years [Fig. 1(A)]. In particular, in both years two peaks were recorded, one in late April–early May and the other in late May [Fig. 1(A)]. Field observations during sampling and comments from farmers in the sampled pistachio fields indicated that beetles showed a clear preference for young leaves through the whole period, as long as they are available. Proportions of males over females varied across the periods within years, although trends showed some differences between years. In 2020, females were much more abundant than males particularly in late May–early June, whereas males seemed more abundant in late April–early May. In 2020, the shift of sex ratio as observed in the field seemed to occur after the peak egg‐deposition period, because egg clusters were recorded essentially in the first ten days of May [Fig. 1(A)]. However, in 2021, females were much more abundant in late April–early May, whereas males were more abundant in late May–early June. In 2021, egg clusters were observed across the whole period with no clear peaks, although they were relatively more abundant on trees during May, as it occurred in 2020. Mating activity also occurred through the whole study period of 2021, especially in the second half of May [Fig. 1(A)].

The 2021 data also revealed that beetles occurred across individual trees in a clumped pattern; that is, few trees had high numbers of collected males or females (>50: 5–10% of trees) whereas most trees had few numbers of collected males or females (<15: 52–66% of trees), and a moderate number had intermediate values (16–49: 29–37% of trees) [Fig. 1(B)]. Mating pairs also were detected, as a consequence, in a similar pattern: 2% of trees had >50 copulae, 17% of trees had 16–49 copulae and 81% of trees had >50 copulae [Fig. 1(B)]. The distribution of egg clusters across trees also seemed likewise clumped, with numbers ranging from zero to seven per tree [Fig. 1(B)]. Indeed, all of the Shapiro–Wilk tests showed lack of normality in these distributions (males: W = 0.79, females: W = 0.79, copulae: W = 0.78, egg clusters: W = 0.85; all *P* < 0.0001), and a moderate (0.89 for egg clusters) to high (males: 2.14, females: 2.12, copulae: 2.26) left skewness.

Our own records and previous records overall showed that, in the Iberian Peninsula, *L. lusitanica* currently occurs in many regions across both East–West and North–South axes [Fig. 2(A); Supporting information Table S1). The species seems to occur mostly in the eastern and southern parts of the Peninsula, and more often in areas not far from the coasts. The west side of the Peninsula, roughly at the border between Spain and Portugal, also concentrates a high number of records, whereas records are scarce in Portugal [Fig. 2(A)].

### 3.2 Climatic niche and potential distribution

Significant differences emerged for all selected BioClim variables between presence points and points with no records [Fig. 2(C)]. In particular, *L. lusitanica* seems to prefer areas with lower day‐to‐night temperature oscillations (BIO3) (Student's *t*‐test: *t* = 32.81, df = 1, *P* = 0.0012), higher continentality (BIO7) (*t* = 16.25, df = 1, *P* < 0.0001), higher temperature of warmest quarter (BIO10) (*t* = 17.44, df = 1, *P* < 0.0001), greater precipitation seasonality (BIO15) (*t* = 11.76, df = 1, *P* < 0.0001) and lower precipitation (BIO18, BIO19) (*t* = 21.05 to 70.75, df = 1, *P* < 0.001) [Fig. 2(C)]. The contribution of each of the variables on the six employed models is shown in Table 1.

**Table 1:** Contributions of the BioClim variables to the six performed models

	BIO10	BIO15	BIO18	BIO19	BIO3	BIO7
GLM	6%	19%	92%	0%	0%	12%
ANN	19%	24%	58%	56%	9%	26%
CTA	13%	3%	32%	39%	11%	30%
RF	4%	5%	12%	6%	5%	33%
MAXENT	25%	6%	23%	16%	11%	30%
GAM	2%	5%	43%	44%	16%	6%

**Figure 3:**
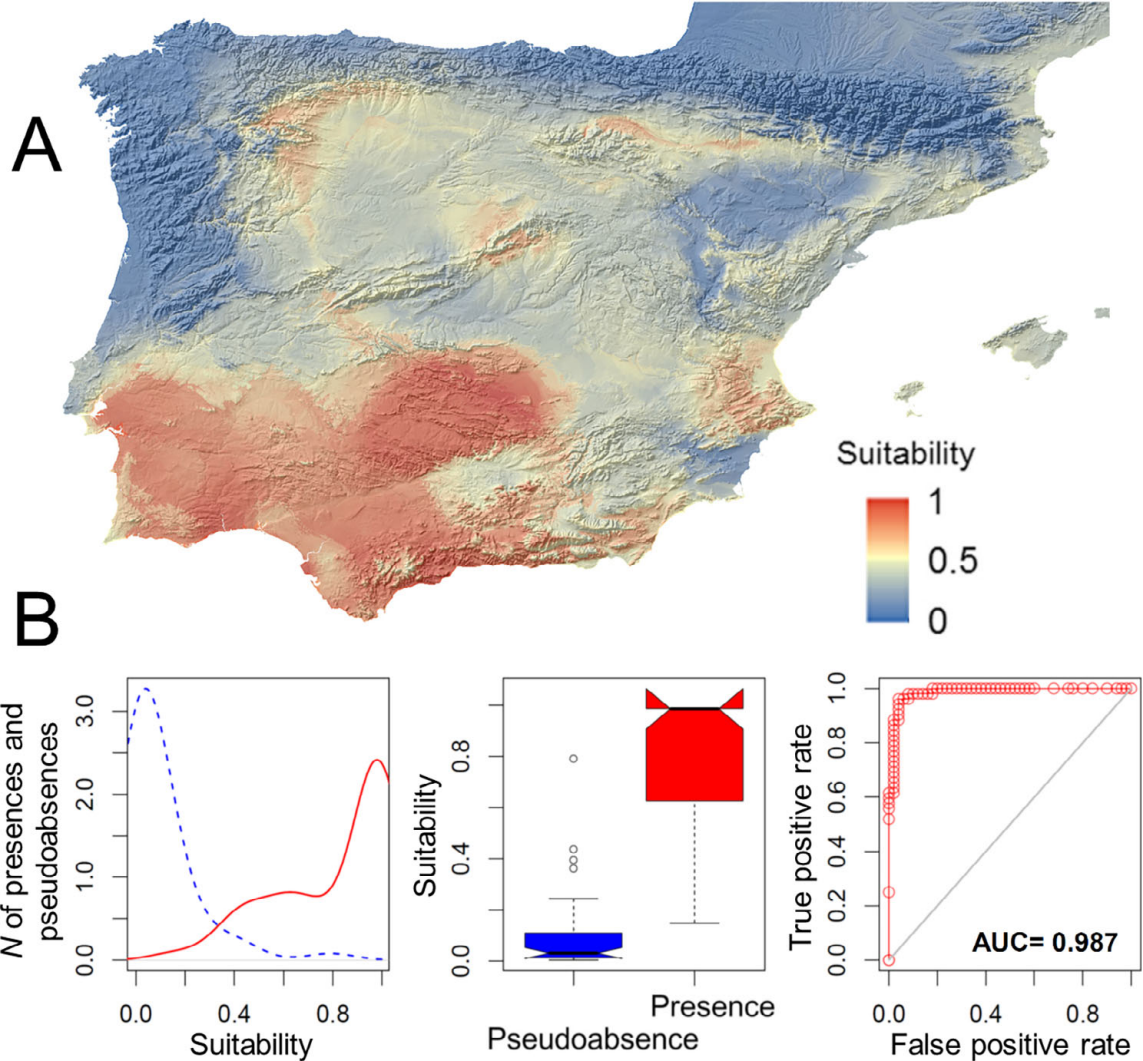
(A) Map showing the predicted potential distribution of *L. lusitanica* in the Iberian Peninsula through the average consensus models. The degree of suitability for the species survival (increasing from blue to red) is shown. (B) Accumulation of number of presences (red continuous line) and number of pseudoabsences (blue dashed line) (in *y*‐axis) considering the suitability of the consensus model of *L. lusitanica* (in *x*‐axis) (left); boxplots of presences (in red) and pseudoabsences (in blue) considering the suitability of the consensus model of *L. lusitanica* (in *y*‐axis) (centre); receiver operating characteristic (ROC) plots for the potential distribution consensus model of *L. lusitanica* (right); the ‘Area under ROC curve’ is the AUC value.

The climatic preferences of *L. lusitanica* gave a suitability map through our modelling procedure [Fig. 3(A)]. The average consensus model had a high performance [Fig. 3(B)], as had the lower and upper models (all AUC > 0.98) (Fig. S1). All of the models, taken individually, showed good AUC and accuracy values (>0.70). Although the RF model tended to over‐fit the model with the highest AUC and accuracy (Fig. S2), in the ensemble modelling procedure this effect was clearly mitigated (Supporting Information, Fig. S2). The average model revealed that the beetle species finds climatically highly suitable areas (>0.8) in the central‐south part of the Iberian Peninsula, especially in the west side; regions with moderate suitability (0.5–0.8) also fall in limited, confined areas of the centre, east (also on the its southern coasts) and north [Fig. 3(A)]. Coastal areas on the west and north side seem especially unsuitable for the species, together with some areas on the north‐east side [Fig. 3(A)]. Of 18 occurrence points recorded through the 2021 sampling, kept for *a posteriori* validation of the model, 15 fell in the predicted highly suitable areas and three in areas with low suitability (0.3) [Table S1; Fig. 2(A)].

**Figure 4:**
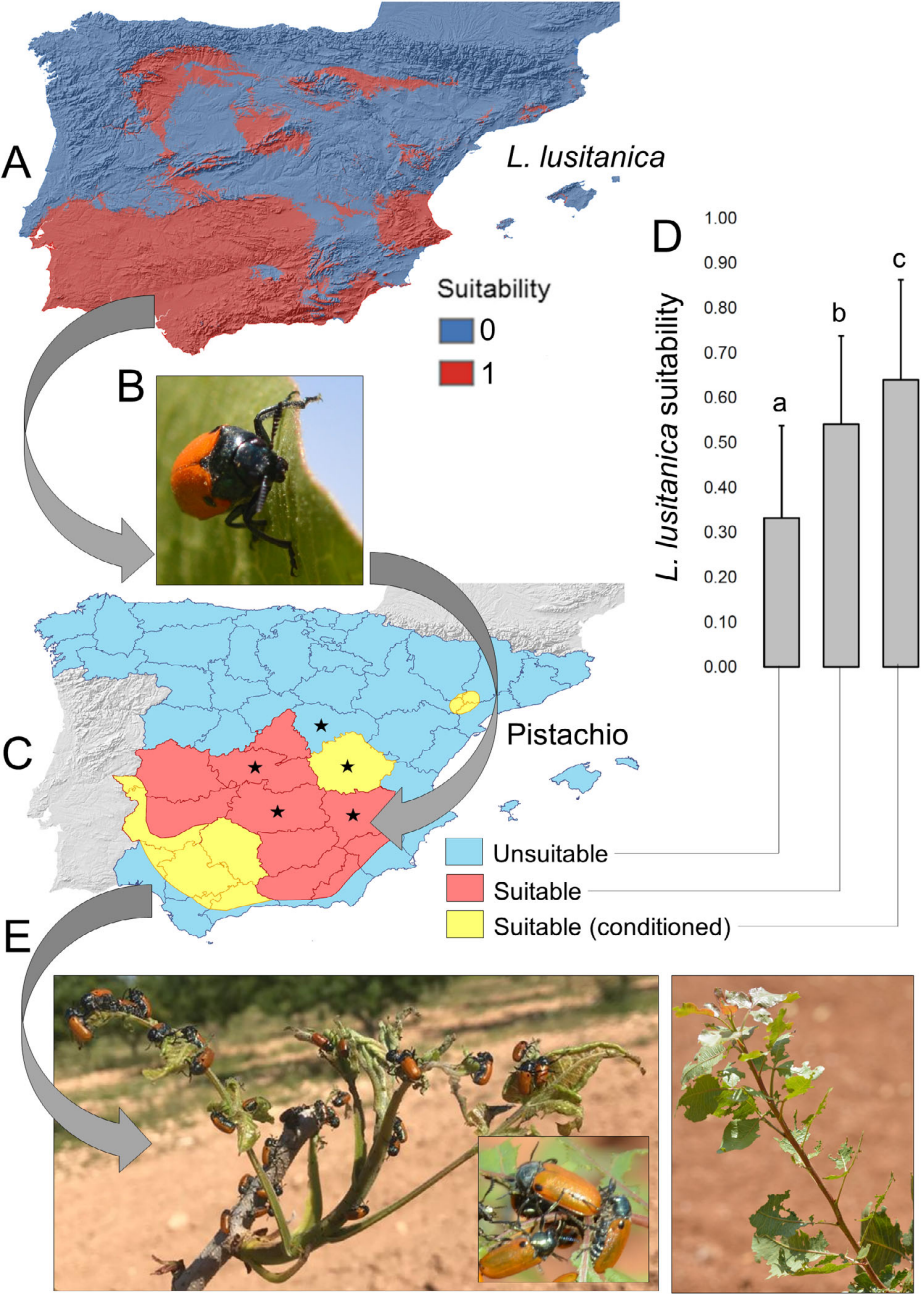
(A) Map showing the predicted potential distribution of *L. lusitanica* in the Iberian Peninsula through the average consensus models, using the cut‐off value for suitability (0.55). (B) A *L. lusitanica* individual on a pistachio leaf. (C) Map showing the suitability of climate for pistachio cultivation across Spain (adapted from Couceiro *et al*.^8^) (stars indicate the provinces belonging to Castilla‐La Mancha). Conditioned suitable areas are those areas in which pistachio will grow satisfactorily only if sown early in Spring. (D) Average values and SEs of *L. lusitanica* across the three pistachio suitability categories (letters represent significant Bonferroni *post hoc* pairwise differences among groups after an ANOVA test). In the figure, curved grey arrows show areas of high beetle suitability corresponding to suitable areas for the crop, which suggests that damage to pistachio crops will likely be stronger in such overlapping areas, as exemplified here (E) by two pictures showing dense clusters of *L. lusitanica* individuals on a single pistachio tree (left) and a heavily affected tree after the attack by *L. lusitanica* (right).

The retrieved map of the suitable areas for pistachio cultivation [Fig. 4(A)] showed an important overlap with the suitable areas (i.e. suitability higher than the cut‐off of 0.55) for *L lusitanica* [Fig. 4(A)]. Hence, in pistachio‐suitable areas, the occurrence of this beetle species [Fig. 4(B)] is very likely in pistachio fields. Furthermore, *L. lusitanica* suitability was highest in areas with ‘conditioned’ suitability for pistachio [i.e. areas that are suitable for early spring cultivars, not for all cultivars, because spring cultivars have fewer crop heat units (CHU), a commonly used metric to quantify the effect of temperature on crop development^55^: 0.640 ± 0.0007], followed by suitable areas with no such restrictions (0.542 ± 0.0005) and finally by unsuitable areas for the crop, where beetle suitability dropped, on average, well below the suitability cut‐off value (0.55) (0.332 ± 0.0003) [Fig. 4(D)]. These three mean suitability values differed significantly (ANOVA test: *F* = 130 913, df = 2, *P* < 0.001; Bonferroni post‐hoc tests for paired comparisons: *P* < 0.001 in all cases). Hence, the overlapping pistachio beetle high suitability areas would lead to an increased impact on the crop by *L. lusitanica* (E).

### 3.3 Survey

All of the contacted farmers send back the answers of the survey, but only 165 questionnaires were valid for our analyses, because we excluded all those reporting *L. lusitanica* presence despite any clear confirmation through the provided pictures and from locations where this leaf beetle species was never reported. Most of the completed surveys (123) came from Castilla‐La Mancha across three provinces (Albacete, Cuenca and Ciudad Real), whereas 30 came from Andalusia and the rest spanned the regions of Castilla y Leon, Madrid and Aragon (Table S2). Hence, the majority of survey data come from the central‐south part of the country, which corresponds with the area most suitable for pistachio cultivation. The areas of the associated pistachio fields ranged from 0.7 to 119 ha in size (11 ± 1.3). The age of the fields was variable: 42 only included very young trees (<3 years), 49 only moderate‐aged trees (3–8 years) and only 14 exclusively >8‐year‐old trees. The large majority of fields (136) did not present any >8‐year‐old trees. The rest of locations showed a mixed component of young and old trees (Table S2).

**Figure 5:**
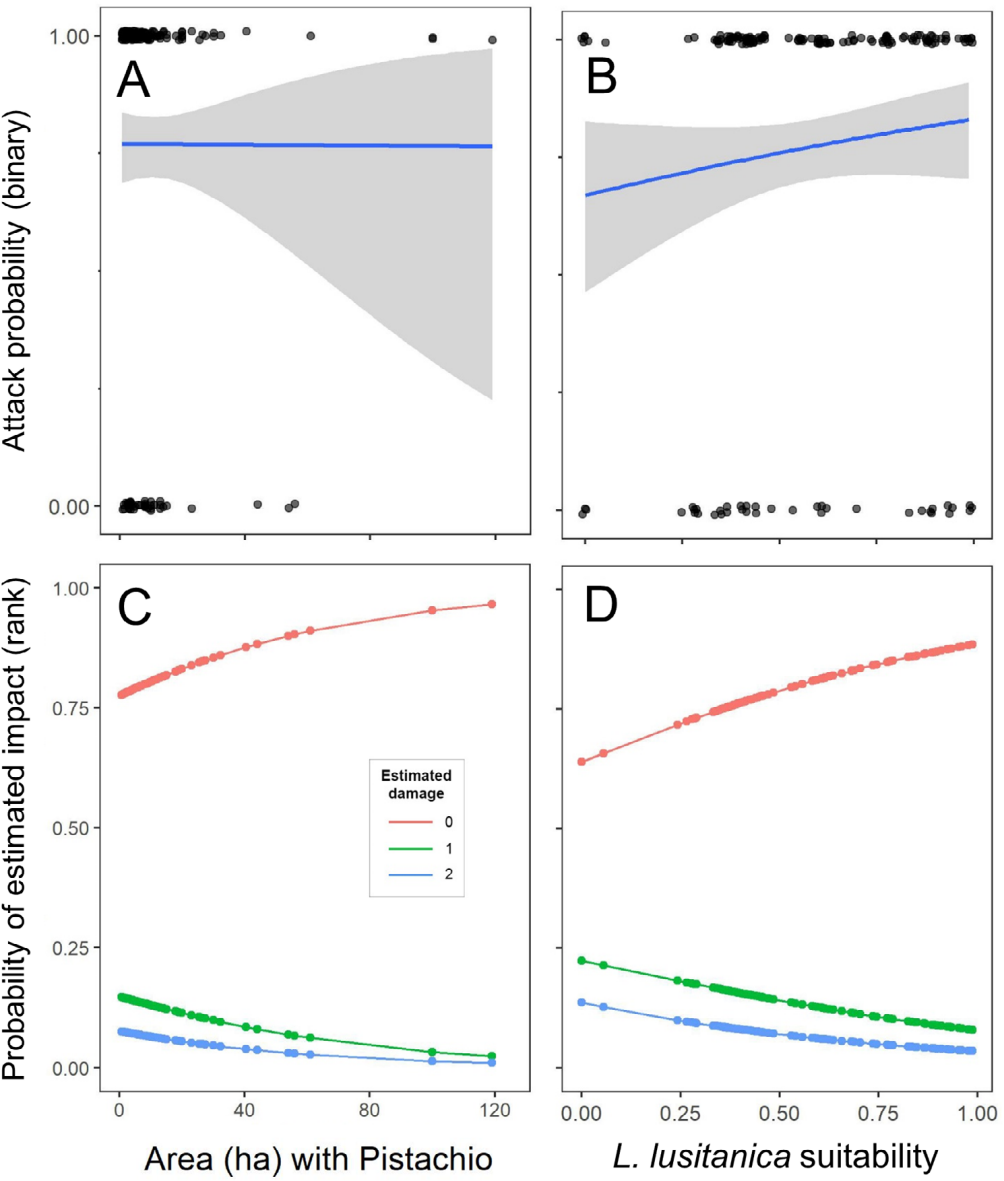
(A,B) Relationships between attack probability by *L. lusitanica* (binary response from the survey) and (A) pistachio field area and (B) *L. lusitanica* suitability for the areas where fields are located; trend lines represent the adjustments to binary logistic regressions, points are the observed values. (C,D) Relationships between the probability of estimated impact (perceived tree loss) resulting from *L. lusitanica* (ranked answer from the survey) and (A) pistachio field area and (B) *L. lusitanica* suitability for the areas where fields are located; trend lines represent the adjustments to ordered logistic regressions, points are the predicted values. In A and B, grey intervals represent the 95% confidence envelops.

Our analysis revealed that both attack probability (i.e. answer to yes/no detection) and perceived impact (i.e. answer to estimated % rank tree loss) by *L. lusitanica* were not affected by field size and % of young, moderate‐aged and old trees in the field [Table 2; Fig. 5(A)–(D)]. A weak, albeit not significant, tendency of a higher attack probability in the most suitable areas for the beetle can at least be suggested [Table 2; Fig. 5(B)]. However, the perceived impact, albeit not significant, seemed to have a weak tendency to decrease in more suitable areas for the beetle [Table 2; Fig. 5(D)].

**Table 2:** Results of the survey to farmers

**Attack probability (binary)**				
**Binary logistic regression**				
**Variable**	**χ** ^**2**^	**df**	***D*** ^**2**^	***P***
Total cultivated area (ha)	0.0006	1	0	0.98
0–3 year‐old % cultivated area	0.18	1	0.001	0.65
4–8 year‐old % cultivated area	0.68	1	0.003	0.41
>8 year‐old % cultivated area	0.26	1	0.001	0.65
*L. lusitanica* suitability	1.54	1	0.008	0.21
**Estimated impact (ranked)**				
**Ordered logistic regression**				
**Variable**	***t***	**df**	***D*** ^**2**^	***P***
Total cultivated area (ha)	−0.94	2	<0.01	0.34
0–3 year‐old % cultivated area	−1.22	2	0.01	0.22
4–8 year‐old % cultivated area	1.01	2	<0.01	0.31
>8 year‐old % cultivated area	−0.45	2	<0.01	0.65
*L. lusitanica* suitability	−1.69	2	0.01	0.09

The Binary logistic regression and the Ordered logistic regression were used to test if attack probability and estimated impact by *L. lusitanica* (respectively) depend on area and age of the pistachio field, and on the climatic suitability of the pistachio field location for *L. lusitanica*.

## 4 DISCUSSION

Ecological and economic damage caused by insect pests which invade recently introduced crop species have to be monitored in detail, because with time they can become devastating at a regional or national scale.^56^, ^57^ Hence, mapping pest species distributions, and predicting which areas that are more likely to suffer the negative effect of such species are relevant to plan future control strategies.^58^, ^59^, ^60^ Our studied crop, pistachio, serves as host to a diverse range of insects and mites, some of them known to strongly impact on trees and hence nut production.^10^, ^61^ In general, plant‐sap‐feeding insects, such as psyllids, plant bugs, mealybugs, stink bugs, scale insects and leafhoppers (all Hemiptera), are considered to be the major pests of this crop globally, with additional injurious species including members of Lepidoptera, Hymenoptera and Coleoptera.^9^, ^10^, ^61^ However, most of our knowledge on pistachio pests comes from countries in which this plant traditionally is cultivated, such as Middle East countries.^10^ Where pistachio was imported relatively recently, information on the diversity, ecology and impact of pests is generally scarcer, and attention of farmers and researchers increased only after damages became apparent and worrying (e.g. in California).^62^ In Spain, pistachio arrived in the 1980s, and studies on its current and potential pests are essentially missing. We present here the first distributional and ecological data on the leaf beetle *L. lusitanica*, a species recently suspected to have an important pest potential for pistachio crops in Spain.^5^

### 4.1 Activity and current distribution

By actualizing the current distribution of *L. lusitanica* in the Iberian Peninsula it appears that the species occurs mostly in the eastern and southern parts of the Peninsula (and western areas of Spain), and more often in areas not far from the coasts. This largely corresponds to a hot and dry Mediterranean climate characterized by lower temperature oscillations, higher temperatures, greater precipitation seasonality and lower precipitation. Temperature is the most important environmental factor that affects insect distribution,^63^, ^64^, ^65^ because its variation has strong consequences on population dynamics, and individuals' physiology, behaviour and interactions with other species (including host plants for pest insects).^63^, ^66^ In Europe, higher temperatures are likely to promote range expansion towards northern latitudes and higher elevations for some pest insects, such as defoliating moths and bark beetles.^67^, ^68^, ^69^ This seems especially true for the southern Mediterranean region, which is at risk not only of increased in temperatures but also of frequency of drought events.^70^, ^71^ Hence, *L. lusitanica*, which at the moment does not find very suitable areas in the central‐northern part of the Iberian Peninsula (except some limited areas), could move to such areas in a drier and hotter future. Such a distributional shift was seen recently in other leaf beetles species. For example, *Chrysolina (Chrysolinopsis) americana* (Linnaeus, 1758), a pest of Lamiaceae with economic importance, such as *Lavandula* spp., *Salvia rosmarinus* and *Thymus* spp., is native to the Mediterranean region, and has experienced an expansion of its distribution in the last few decades, especially to new regions in the north and eastern Mediterranean basin.^72^ Additionally, such a possible shift or even widening in the distribution of *L. lusitanica* may be accompanied in the future by the arrival of alien leaf beetle species from outside the Iberian Peninsula, favoured by climate change and globalization of trade. Indeed, some of the leaf beetles known to be injurious pests of crops and ornamental plants have already established in Europe in recent times.^73^

Although we found similar overall phenology of the species in both 2020 and 2021, with two main peaks of activity (late April–early May and late May), peaks of females and males differed in the two study years, suggesting that populations may adjust the sex ratio in response of varying environmental factors. It is also possible that the different sampling procedures in the two years [several locations nonuniformly sampled (2020) *versus* structured sampling at one location (2021)] may account for such a difference. Egg laying, instead, seemed in both years mainly – although not exclusively – to fall during May, roughly corresponding to a shift in adult sex‐ratio. The 2021 data also reveal that mating seemed essentially continuous through the whole activity period. Hence, the overall phenological/activity picture observed in 2020 was roughly confirmed by the new data of 2021. These phenological data show that pistachio trees are vulnerable also when they are young, or when new leaves are produced, mostly in early spring. These results agree with what was suggested by the Ministerio de Agricultura y Pesca, Alimentación y Medio Ambiente of Spain (MAPAMA, now MAPA), that special attention should be paid to this pest as a consequence of the considerable damage that it can cause in a few hours, especially in the youngest plantations or in the most tender leaves of adult trees. Even at a regional level, a recent report highlights the importance of monitoring this species in detail owing to its quick and high‐density appearance in Pistachio fields (Consejería de Agricultura, Agua y Desarrollo Rural de Castilla‐La Mancha^74^). MAPA recommends, in young plantations, that visual surveillance be carried out every few days.^5^

### 4.2 Potential distribution

Our model shows that *L. lusitanica* already occupies all potential range areas within its current distribution in the Iberian Peninsula. Therefore, apparently no new areas would remain colonizable by this species in this territory, at least under the current climate. Several studies analyzed the potential distribution of species of leaf beetles and the climatic factors most important in their distribution patterns. For example, Iannella *et al*.^75^ found that suitable climatic conditions for the invasive *Luperomorpha xanthodera* (Fairmaire, 1888) (which is a pest of ornamental plants) are similar to those in areas where this species currently occurs, which includes Ireland and some Balkan countries, where the species is not yet recorded. The authors predict a northeastern expansion in the future, spanning many countries currently lacking suitable climatic conditions for this species, in agreement with the increase of the mean temperature in the coldest quarter of the year, which is predicted to rise over the next 30 years. However, Freeman *et al*.^76^ studied the potential distribution of *L. lilii* (Scopoli, 1793), an invasive pest of cultivated and native lilies (Liliaceae) native to Europe and Asia, and introduced to North America in the 1940s, finding that the beetle should be able to establish throughout the range of most North American Liliaceae, including species of special conservation concern. The authors identified in that case precipitation in the driest quarter and annual average temperatures as the factors most strongly affecting *L. lilii* distribution, with the species performing poorly in very dry, hot or cold environments.

Interestingly, our distribution model predicts that *L. lusitanica* would find optimal climatic conditions largely where pistachio also has suitable conditions for growth. Hence, *L. lusitanica* has a great potential to establish as a pest in such pistachio‐growing areas. This potential could be even greater when considering that this beetle species is polyphagous^18^ and in Spain also attacks other crops (grapevine, plum tree, avocado^20^) whose cultivated areas, in particular for grapevine, extend through most of Castilla‐La Mancha (http://pagina.jccm.es/ivicam/servicios/mapaviti.html) and for avocado in some interior parts of Andalusia (http://www.avocadosource.com/temp/OLD%20WAC%20II/WAC2_p647.htm), also overlap with those that are very suitable for pistachio cultivation. Hence, *L. lusitanica* in those areas would have more than one host crop species available, likely favouring its persistence. Furthermore, the highest suitability for the pest beetle was detected in areas that are ‘conditioned’ suitable for pistachio, compared with the areas overall suitable without special restrictions. Such ‘conditioned’ areas largely are those where only those pistachio cultivars with moderate CHU would grow earlier in spring, and this is a period that would probably already find *L. lusitanica* ready to attack it.

### 4.3 Surveys

Survey results agreed with those obtained by the SDM, because we found a weak tendency for a higher attack probability in the most suitable areas for the beetle. However, unexpectedly, the perceived impact weakly tended to be lower in more suitable areas for the beetle, suggesting that other factors may shape its pest potential in a complex way. Among these factors, we may exclude field area and age, which did not influence the survey answers. Furthermore, our results from the surveys, which failed to clearly associate crop field characteristics with tree damage by beetles, may depend on the strong preference for certain trees over others within fields, leading to the observed aggregated pattern. Indeed, our sampling showed that few trees accumulate most of the collected individuals, with most of the trees having only few or even no beetles observed. Hence, if farmers inspected their trees by random to respond to the survey, they may have mainly inspected those trees with very few beetles and thus low or no damage.

Despite the claimed high vulnerability of young plantations by reports of public administrations (e.g. Ministry), our results from the survey to pistachio growers failed to show that attack probability and perceived impact are associated with young pistachio fields (0–3 years old), compared with other ages. Our survey results may indeed reflect that rather than whole‐field age, it is the presence of young leaves on trees of every age that may be conditioning the vulnerability of pistachio to this pest.

### 4.4 Limitations of the study

Our study has limitations that depend mainly on the nature of the data themselves and on the interactions among all the factors shaping pest distribution and impact. On the one hand, although occurrence records are certainly reliable, because they come from direct observations and correct identification of the pest species, species absences in our study are pseudoabsences. This means that a species not being reported from an area does not necessarily mean that is actually absent from that area.^41^ Hence, records of true absence would improve the precision of the models used here. On the other hand, we modelled the potential distribution of *L. lusitanica* based exclusively on climatic variables, whereas it is clear that, in the interactions of insect pests and their host plants, other abiotic as well as biotic factors, such as water supply, soil chemical properties, competition with other insect species and parasitism by natural enemies, are important (reviewed in Tonnang *et al*.^77^). By using only one portion of the fundamental niche of the species, that is, climatic conditions, we are not taking into account biotic (as well as additional abiotic) drivers of distributions in the models (e.g. food resources, interactions with other species, habitat type, land cover).^78^, ^79^ Furthermore, physiological experiments devoted to ascertain which conditions the species are actually able to tolerate will add accuracy to any prediction on potential distribution.^41^, ^79^

## 5 CONCLUSION

Our results overall provide data that suggest an important pest potential of *L. lusitanica* in the Iberian Peninsula. The possible risk of relevant damage by this pest, particularly in the context of strong extension in pistachio cultivation in Spain and ongoing climate change, is in line with what has been observed for other *Labidostomis* species on pistachio in other Mediterranean countries. Indeed, both *L. diversifrons* and *L. longimana* have been reported in Syria and Turkey (respectively) causing important injuries to the leaves and consequently making the trees weaker.^21^, ^22^ Hence, further investigations are needed on *L. lusitanica* to help planning of efficient prevention and control strategies. At the moment, the MAPA recommends a foliar spray treatment (Lambda cyhalothrin 5% at a dose of 0.02–0.003%, and a maximum of 1.5 kg ha^–1^) if this pest beetle is detected on pistachio trees.^7^ However, such a control method is inadequate for ecological production of pistachio, for which no products currently are authorized by MAPA to treat this pest. Biological control may be an alternative, although data in the case of *L. lusitanica* are lacking. On other crops (e.g. beans, cucumbers), natural enemies of leaf beetles with biocontrol potential especially include predatory mirid bugs (Heteroptera) which feed on beetle eggs.^80^ Other biological control strategies are related to entomopathogenic nematodes and fungi.^81^ However, none of these potential control agents are species‐specific natural enemies, implying that they may also attack nontarget species in the crop, certainly a risky scenario for the nonpestiferous fauna. Hence, it would be important to conduct and invest in studies on the possible natural enemies with high specificity that could be used as successful biocontrol agents of *L. lusitanica*.

## CONFLICT OF INTEREST

The authors declare that they have no conflict of interest.

## Supporting information


**Figure S1.** Maps showing the predicted potential distribution of *L. lusitanica* in the Iberian Peninsula through our upper (A) and lower (B) consensus model based on BioClim variables. The degree of suitability for the species survival (increasing from blue to red) is shown.

**Figure S2.** Relationship between ROC values and accuracy for the six modelling procedures of potential distribution of *L. lusitanica*, showing that the effect to over‐fit the model typical of the RF procedure is mitigated in the ensemble modelling used.

**Table S1.** Georeferenced records of *L. lusitanica* in the Iberian Peninsula (ordered by increasing latitude). GBIF, Global Biodiversity Information Facility; BV, Biodiversidad Virtual website; iN, iNaturalist website.

**Table S2.** Data retrieved from the surveys sent to pistachio growers, with fields ordered by increasing total area. Attack probability is 0 or 1 if farmers report no or at least one observation of *L. lusitanica* on the trees. Estimated impact (only if answer was 1 for attack probability) was evaluated by the farmers choosing one of three tanks of perceived tree loss (0 = no loss, 1 = <10% loss, 2 = >10% loss). NA, not applicable; ‐, no answer was provided.

## Data Availability

The data are available in Tables and in the Supplementary files Table S1 and Table S2.
