# Supplementary figures and images for: The leaf beetle Labidostomis lusitanica (Coleoptera: Chrysomelidae) as an Iberian pistachio pest: projecting risky areas
Source: Pest Manag Sci. 2021 Sep 16;78(1):217–29. doi: 10.1002/ps.6624 (PMC9293163; doi:10.1002/ps.6624)

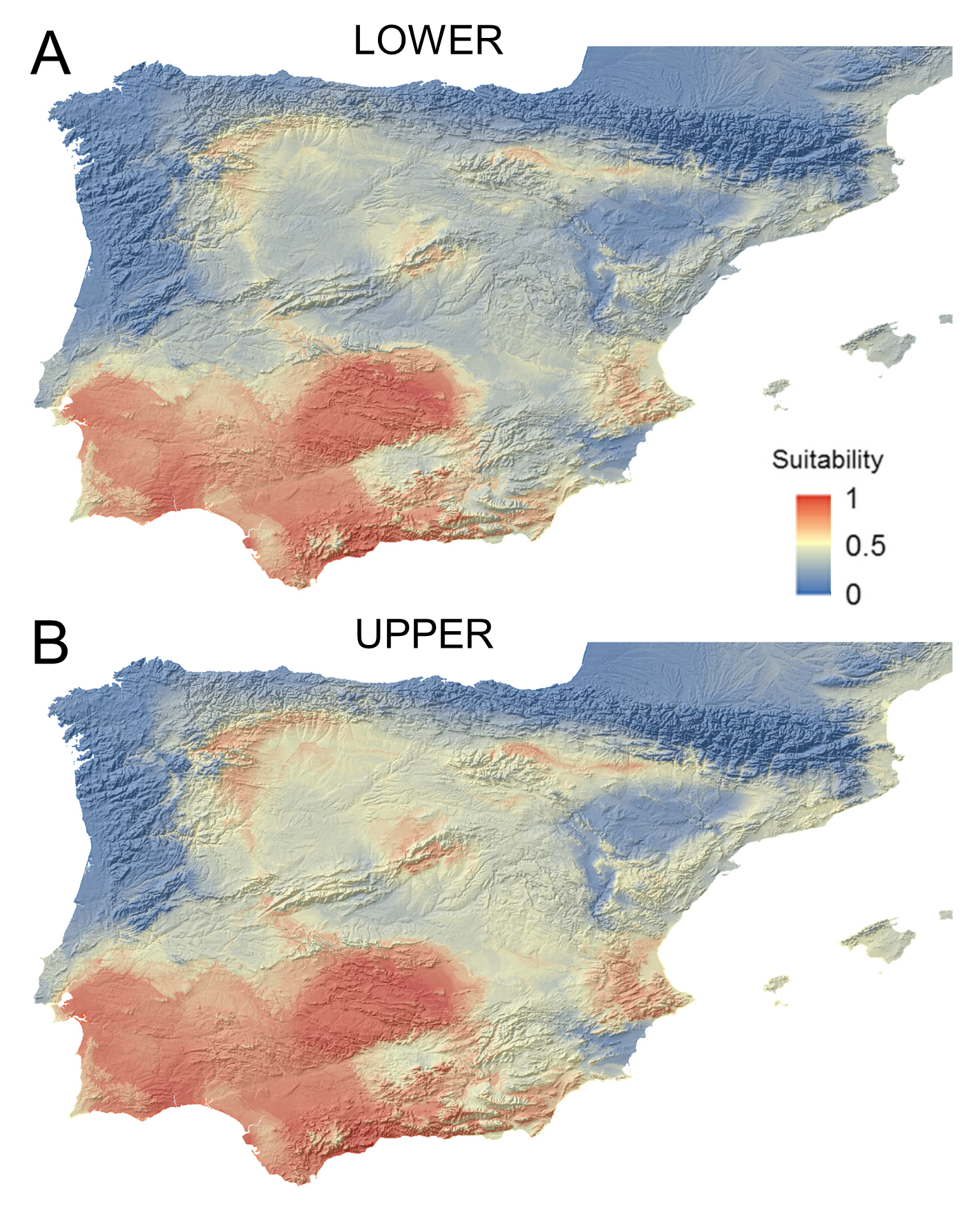

Supplement: Supplementary file 1 — Figure S1. Maps showing the predicted potential distribution of L. lusitanica in the Iberian Peninsula through our upper (A) and lower (B) consensus model based on BioClim variables. The degree of suitability for the species survival (increasing from blue to red) is shown. [file PS-78-217-s003.tif]

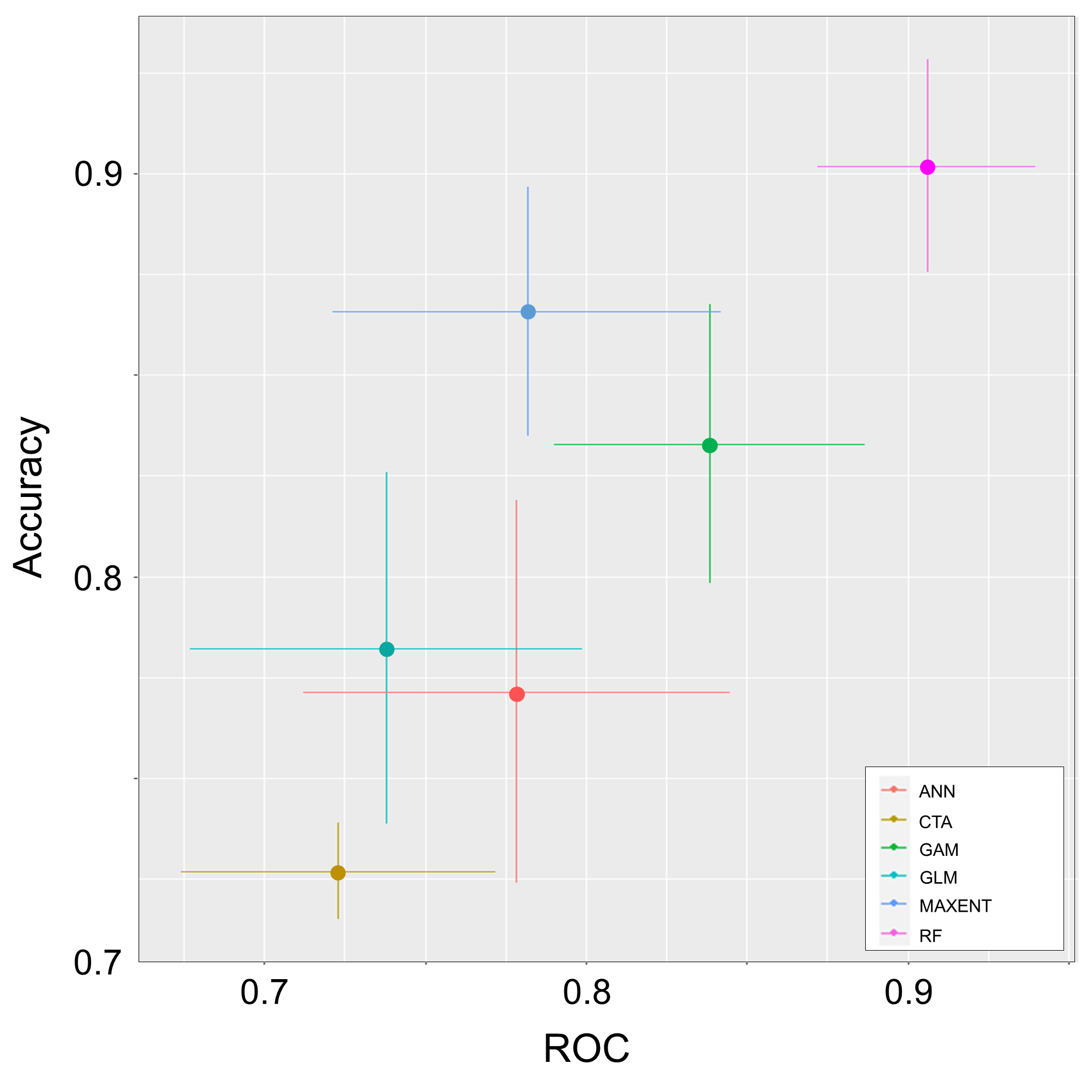

Supplement: Supplementary file 2 — Figure S2. Relationship between ROC values and accuracy for the six modelling procedures of potential distribution of L. lusitanica, showing that the effect to over‐fit the model typical of the RF procedure is mitigated in the ensemble modelling used. [file PS-78-217-s004.tif]
