# Supplementary material for: The leaf beetle Labidostomis lusitanica (Coleoptera: Chrysomelidae) as an Iberian pistachio pest: projecting risky areas
Source: Pest Manag Sci. 2021 Sep 16;78(1):217–29. doi: 10.1002/ps.6624 (PMC9293163; doi:10.1002/ps.6624)
Supplement: Supplementary file 3 — Table S1. Georeferenced records of L. lusitanica in the Iberian Peninsula (ordered by increasing latitude). GBIF, Global Biodiversity Information Facility; BV, Biodiversidad Virtual website; iN, iNaturalist website. [file PS-78-217-s002.docx]

**Table S1.** Georeferenced records of *Labidostomis lusitanica* in the Iberian Peninsula (ordered by increasing latitude). GBIF = Global Biodiversity Information Facility, BV = Biodiversidad Virtual website, iN = iNaturalist website. * indicates that the record is from the 2021’s sampling and that was used to validate the potential distribution model based on the all the other records.

| **ID** | **Latitude** | **Longitude** | **Source** |
| --- | --- | --- | --- |
| 1 | 36.139 | -5.456 | Petitpierre et al. (2011) |
| 2 | 36.51 | -4.89 | Vela et al. (2014) |
| 3 | 36.605 | -5.801 | Petitpierre et al. (2011) |
| 4 | 36.75 | -5.8 | GBIF |
| 5 | 36.774 | -3.684 | Vela et al. (2017) |
| 6 | 36.785 | -3.852 | Vela et al. (2017) |
| 7 | 36.854 | -4.024 | Vela et al. (2017) |
| 8 | 36.858 | -2.777 | BV |
| 9 | 36.86 | -4.033 | Vela et al. (2017) |
| 10 | 36.897 | -4.043 | Vela et al. (2017) |
| 11 | 36.909 | -4.76 | Doguet et al. (1996) |
| 12 | 36.911 | -2.458 | BV |
| 13 | 37 | -4 | GBIF |
| 14 | 37.055 | -7.983 | iN |
| 15 | 37.122 | -3.447 | Petitpierre & Daccordi (2013) |
| 16 | 37.181 | -5.935 | BV |
| 17 | 37.308 | -6.597 | Petitpierre & López-Pérez (2015) |
| 18 | 37.316 | -6.842 | Petitpierre & López-Pérez (2015) |
| 19 | 37.383 | -5.996 | BV |
| 20 | 37.384 | -6.838 | Petitpierre & López-Pérez (2015) |
| 21 | 37.393 | -7.81 | GBIF |
| 22 | 37.424 | -4.18 | Daccordi & Petitpierre (1977) |
| 23 | 37.521 | -7.417 | Petitpierre & López-Pérez (2015) |
| 24 | 37.901 | -2.939 | Daccordi & Petitpierre (1977) |
| 25 | 37.92 | -2.992 | Daccordi & Petitpierre (1977) |
| 26 | 37.955 | -2.67 | Petitpierre & Daccordi (2013) |
| 27 | 38.068 | -3.35 | BV |
| 28 | 38.2 | -3.5 | This study* |
| 29 | 38.6 | -0.8 | GBIF |
| 30 | 38.603 | -0.804 | BV |
| 31 | 38.609 | -0.804 | BV |
| 32 | 38.619 | -3.39 | This study |
| 33 | 38.619 | -3.39 | This study |
| 34 | 38.619 | -3.39 | This study |
| 35 | 38.619 | -3.39 | This study |
| 36 | 38.619 | -3.39 | This study |
| 37 | 38.619 | -3.39 | This study |
| 38 | 38.619 | -3.39 | This study |
| 39 | 38.619 | -3.39 | This study |
| 40 | 38.619 | -3.39 | This study |
| 41 | 38.619 | -3.39 | This study |
| 42 | 38.619 | -3.39 | This study |
| 43 | 38.619 | -3.39 | This study |
| 44 | 38.619 | -3.39 | This study |
| 45 | 38.619 | -3.39 | This study |
| 46 | 38.619 | -3.39 | This study |
| 47 | 38.619 | -3.391 | This study |
| 48 | 38.619 | -3.39 | This study |
| 49 | 38.619 | -3.39 | This study |
| 50 | 38.619 | -3.39 | This study |
| 51 | 38.619 | -3.39 | This study |
| 52 | 38.619 | -3.39 | This study |
| 53 | 38.619 | -3.39 | This study |
| 54 | 38.619 | -3.39 | This study |
| 55 | 38.619 | -3.39 | This study |
| 56 | 38.619 | -3.39 | This study |
| 57 | 38.619 | -3.391 | This study |
| 58 | 38.619 | -3.391 | This study |
| 59 | 38.619 | -3.39 | This study |
| 60 | 38.619 | -3.39 | This study |
| 61 | 38.619 | -3.39 | This study |
| 62 | 38.619 | -3.39 | This study |
| 63 | 38.619 | -3.39 | This study |
| 64 | 38.62 | -3.39 | This study |
| 65 | 38.62 | -3.391 | This study |
| 66 | 38.62 | -3.39 | This study |
| 67 | 38.62 | -3.391 | This study |
| 68 | 38.62 | -3.39 | This study |
| 69 | 38.62 | -3.391 | This study |
| 70 | 38.62 | -3.39 | This study |
| 71 | 38.62 | -3.39 | This study |
| 72 | 38.62 | -3.39 | This study |
| 73 | 38.62 | -3.39 | This study |
| 74 | 38.62 | -3.39 | This study |
| 75 | 38.62 | -3.39 | This study |
| 76 | 38.62 | -3.39 | This study |
| 77 | 38.62 | -3.391 | This study |
| 78 | 38.62 | -3.39 | This study |
| 79 | 38.62 | -3.391 | This study |
| 80 | 38.62 | -3.39 | This study |
| 81 | 38.62 | -3.39 | This study |
| 82 | 38.62 | -3.391 | This study |
| 83 | 38.62 | -3.39 | This study |
| 84 | 38.62 | -3.39 | This study |
| 85 | 38.62 | -3.39 | This study |
| 86 | 38.62 | -3.39 | This study |
| 87 | 38.62 | -3.39 | This study |
| 88 | 38.62 | -3.39 | This study |
| 89 | 38.62 | -3.39 | This study |
| 90 | 38.62 | -3.39 | This study |
| 91 | 38.62 | -3.39 | This study |
| 92 | 38.62 | -3.39 | This study |
| 93 | 38.62 | -3.39 | This study |
| 94 | 38.62 | -3.39 | This study |
| 95 | 38.62 | -3.39 | This study |
| 96 | 38.621 | -3.39 | This study |
| 97 | 38.621 | -3.39 | This study |
| 98 | 38.663 | -3.366 | This study* |
| 99 | 38.687 | -3.668 | This study |
| 100 | 38.688 | -3.669 | This study |
| 101 | 38.688 | -3.669 | This study |
| 102 | 38.688 | -3.67 | This study |
| 103 | 38.688 | -3.669 | This study |
| 104 | 38.755 | -8.961 | GBIF |
| 105 | 38.863 | -3.853 | This study |
| 106 | 38.863 | -3.854 | This study |
| 107 | 38.863 | -3.853 | This study |
| 108 | 38.863 | -3.854 | This study |
| 109 | 38.863 | -3.854 | This study |
| 110 | 38.863 | -3.854 | This study |
| 111 | 38.863 | -3.854 | This study |
| 112 | 38.863 | -3.854 | This study |
| 113 | 38.863 | -3.854 | This study |
| 114 | 38.863 | -3.854 | This study |
| 115 | 38.863 | -3.853 | This study |
| 116 | 38.864 | -3.854 | This study |
| 117 | 38.864 | -3.853 | This study |
| 118 | 38.864 | -3.854 | This study |
| 119 | 38.864 | -3.854 | This study |
| 120 | 38.864 | -3.854 | This study |
| 121 | 38.864 | -3.855 | This study |
| 122 | 38.864 | -3.855 | This study |
| 123 | 38.864 | -3.854 | This study |
| 124 | 38.864 | -3.853 | This study |
| 125 | 38.866 | -3.853 | This study |
| 126 | 38.866 | -3.853 | This study |
| 127 | 38.866 | -3.853 | This study |
| 128 | 38.866 | -3.853 | This study |
| 129 | 38.866 | -3.853 | This study |
| 130 | 38.866 | -3.853 | This study |
| 131 | 38.866 | -3.853 | This study |
| 132 | 38.866 | -3.853 | This study |
| 133 | 38.866 | -3.853 | This study |
| 134 | 38.866 | -3.854 | iN |
| 135 | 38.866 | -3.853 | This study |
| 136 | 38.867 | -3.854 | This study |
| 137 | 38.883 | -6.996 | This study* |
| 138 | 38.965 | -3.891 | This study |
| 139 | 38.975 | -3.96 | This study* |
| 140 | 38.976 | -3.873 | This study* |
| 141 | 38.983 | -3.917 | This study |
| 142 | 38.996 | -3.373 | This study* |
| 143 | 39.003 | -3.963 | This study* |
| 144 | 39.062 | -8.178 | GBIF |
| 145 | 39.08 | -1.23 | GBIF |
| 146 | 39.08 | -1.21 | GBIF |
| 147 | 39.12 | -0.97 | GBIF |
| 148 | 39.126 | -3.907 | This study* |
| 149 | 39.016 | -3.75 | This study* |
| 150 | 39.146 | -4.153 | This study* |
| 151 | 39.17 | -0.92 | GBIF |
| 152 | 39.2 | -1.02 | GBIF |
| 153 | 39.216 | -3.6 | This study* |
| 154 | 39.22 | -0.73 | GBIF |
| 155 | 39.283 | -2.316 | This study* |
| 156 | 39.465 | -3.529 | This study* |
| 157 | 39.48 | -1.39 | GBIF |
| 158 | 39.52 | -1.48 | GBIF |
| 159 | 39.539 | -8.864 | iN |
| 160 | 39.595 | -3.046 | This study* |
| 161 | 39.61 | -0.95 | GBIF |
| 162 | 39.733 | -2.917 | This study |
| 163 | 39.803 | -7.46 | Baselga & Novoa (2003) |
| 164 | 39.83 | -0.66 | GBIF |
| 165 | 39.869 | -4.997 | This study* |
| 166 | 39.901 | -3.321 | This study* |
| 167 | 40.293 | -5.09 | García-Ocejo et al. (1992) |
| 168 | 40.323 | -3.108 | This study* |
| 169 | 40.365 | -3.822 | BV |
| 170 | 40.535 | -3.287 | iN |
| 171 | 40.56 | -0.24 | GBIF |
| 172 | 40.575 | -4.006 | García-Orcejo & Gurrea (1995) |
| 173 | 40.576 | -3.929 | García-Orcejo & Gurrea (1995) |
| 174 | 40.67 | -0.1 | GBIF |
| 175 | 40.71 | -3.996 | García-Orcejo & Gurrea (1995) |
| 176 | 40.729 | -4.014 | García-Orcejo & Gurrea (1995) |
| 177 | 40.749 | -3.994 | García-Orcejo & Gurrea (1995) |
| 178 | 40.795 | -3.343 | García-Orcejo & Gurrea (1995) |
| 179 | 40.865 | -3.616 | García-Orcejo & Gurrea (1995) |
| 180 | 40.87 | -3.885 | García-Orcejo & Gurrea (1995) |
| 181 | 40.872 | -3.663 | García-Orcejo & Gurrea (1995) |
| 182 | 40.935 | -1.091 | GBIF |
| 183 | 40.935 | 0.109 | iN |
| 184 | 40.955 | -3.797 | García-Orcejo & Gurrea (1995) |
| 185 | 41.187 | -7.523 | iN |
| 186 | 41.201 | -4.577 | BV |
| 187 | 41.342 | -6.709 | Baselga & Novoa (2003) |
| 188 | 41.346 | -7.28 | GBIF |
| 189 | 41.364 | -6.329 | Baselga & Novoa (2003) |
| 190 | 41.371 | -6.38 | Baselga & Novoa (2003) |
| 191 | 41.473 | 2.209 | GBIF |
| 192 | 41.639 | 2.018 | Echave et al. (2016) |
| 193 | 41.65 | -4.691 | BV |
| 194 | 41.668 | -2.418 | GBIF |
| 195 | 41.695 | 2.005 | Echave et al. (2016) |
| 196 | 41.771 | 2.525 | GBIF |
| 197 | 41.817 | 1.404 | iN |
| 198 | 41.904 | -6.016 | GBIF |
| 199 | 41.913 | 0.96 | BV |
| 200 | 42 | 3 | GBIF |
| 201 | 42.021 | -6.365 | Baselga & Novoa (2002) |
| 202 | 42.112 | -2.674 | Moreno & Sañudo (1999) |
| 203 | 42.177 | -2.705 | Moreno & Sañudo (1999) |
| 204 | 42.178 | -1.751 | Moreno & Sañudo (1999) |
| 205 | 42.263 | -2.763 | Moreno & Sañudo (1999) |
| 206 | 42.318 | -2.76 | Moreno & Sañudo (1999) |
| 207 | 42.33 | -2.487 | Moreno & Sañudo (1999) |
| 208 | 42.334 | -6.807 | Baselga & Novoa (2002) |
| 209 | 42.382 | -6.674 | Petitpierre & Gómez-Zurita (1998) |
| 210 | 42.399 | -2.603 | Moreno & Sañudo (1999) |
| 211 | 42.405 | -2.122 | Moreno & Sañudo (1999) |
| 212 | 42.413 | -2.456 | Moreno & Sañudo (1999) |
| 213 | 42.448 | -6.947 | Baselga & Novoa (2002) |
| 214 | 42.448 | -2.337 | Moreno & Sañudo (1999) |
| 215 | 42.453 | -6.9 | Baselga & Novoa (2002) |
| 216 | 42.463 | -2.445 | Moreno & Sañudo (1999) |
| 217 | 42.503 | -6.933 | Baselga & Novoa (2002) |
| 218 | 42.591 | -6.272 | Petitpierre & Gómez-Zurita (1998) |
| 219 | 42.886 | -5.536 | Petitpierre & Gómez-Zurita (1998) |
| 220 | 42.891 | -5.6 | Petitpierre & Gómez-Zurita (1998) |
| 221 | 42.993 | -5.745 | Petitpierre (2005) |
| 222 | 42.995 | -5.761 | Petitpierre (2005) |
| 223 | 42.997 | -6.42 | Petitpierre (2005) |
| 224 | 43.076 | -4.544 | Petitpierre (2015) |

**References**

Baselga A, Novoa, F (2002) Los Chrysomelidae (Coleoptera) de las sierras orientales de Ourense (Galicia, noroeste de la Península Ibérica). *Boletín de la Asociación española de Entomología* 26(3-4), 57-73.

Baselga A, Novoa, F (2003) Los Chrysomelidae de los Arribes del Duero, noroeste de la Península Ibérica (Coleoptera). *Nouvelle Revue d’Entomologie* (NS) 20(2), 117-131.

BiodiversidadVirtual.org, Insectarium Virtual. *Labidostomis lusitanica.* https://www.biodiversidadvirtual.org/insectarium/Labidostomis-(L.)-lusitanica-(Germar-1824)-cat15073.html [accessed 17 November 2020]

Daccordi M, Petitpierre E (1977) Coleópteros Crisomélidos de la Sierra de Cazorla (Jaén) y descripción de una nueva especie de *Clytra* Laich (Coleoptera, Chrysomelidae). *Miscel· lània Zoològica* 4, 225-235.

Doguet S, Bastazo G, Bergeal M, Vela JM (1996) Contribution à l’étude des Chrysomelidae d’Andalousie (Coleoptera). *Nouvelle Revue d’Entomologie* (NS) 13(4), 315-323.

Echave P, Trocoli S, Bentanachs J (2016) Catálogo de los crisomélidos (Coleoptera: Chrysomelidae) del Parc Natural de Sant Llorenç del Munt i l’Obac (Barcelona, España). *Heteropterus Revista de Entomología* 16(2): 165-177.

García-Ocejo A, Gurrea P (1995) Los crisomélidos (Coleoptera: Chrysomelidae) de la sierra de Guadarrama (España central). Análisis biogeográfico. *Boletin de la Asociacion Espanola de Entomologia* 19(3-4), 51-68.

García-Ocejo A, Gurrea P, Petitpierre E (1992) Chrysomelidae (Coleoptera) de la sierra de Gredos (Sistema Central): datos faunísticos, ecológicos y fenológicos. *Miscel· lània Zoològica*, 16, 81-92.

GBIF.org, *Labidostomis lusitanica* (Germar, 1824) in GBIF Secretariat 2019. GBIF.org GBIF Occurrence Download https://doi.org/10.15468/dl.6zf345 [accessed 17 November 2020]

iNaturalist.org, *Labidostomis lusitanica*. https://www.inaturalist.org/taxa/468896-Labidostomis-lusitanica [accessed 17 November 2020]

Moreno IP, Sañudo FJC (1999) Datos para el catálogo de coleópteros de La Rioja (Insecta, Coleoptera). *Zubía* 11, 93-126.

Petitpierre E, Daccordi M (2013) Chrysomelidae (Coleoptera) de las sierras del Altiplano de Granada (Granada, Andalucía). *Zoologica Baetica* 24, 53-78.

Petitpierre E, Bastazo G, Vela JM (2011) Estudio faunístico de los crisomélidos de la provincia de Cádiz, España (Coleoptera, Chrysomelidae). *Zoologica Baetica* 22, 137-170.

Petitpierre E., Gómez-Zurita J (1998) Los Chrysomelidae de León; NO de España (Coleoptera). *Nouvelle Revue d’Entomologie* (NS) 15, 13-26.

Petitpierre E (2015) Especies nuevas de Chrysomelidae (Coleoptera) para la fauna de Asturias y/o Cantabria. *Boletin de la Asociacion Espanola de Entomologia* 39 (3-4), 275-279.

Petitpierre E, López-Pérez JJ (2015) Catálogo corológico de los crisomélidos (Coleoptera: Chrysomelidae) de la provincia de Huelva (Suroeste de Andalucía, España). *Boletín de la Sociedad Entomológica Aragonesa (S.E.A.)* 56, 221–241.

Petitpierre E (2005). Listado de Chrysomelidae (Coleoptera) de Asturias y Cantabria. *Boletin de la Asociacion Espanola de Entomologia* 29, 51-72.

Vela J, García Méndez-Villamil G, Cuesta A, López Ttrujillo D, Creus E, Gallardo JB (2014) Clytrini (Insecta: Coleoptera: Chrysomelidae) as occasional leaf feeders on avocado and plum trees in Southern Spain. *Boletin de la Asociacion Espanola de Entomologia* 38, 325-330.

Vela JM, Bastazo G, Fritzlar F (2017) Inventario comentado de los crisomélidos (Coleoptera, Chrysomelidae) de las Sierras Tejeda y Almijara y los Acantilados de Maro (Sur de España, Málaga-Granada). *Boletin de la Asociacion Espanola de Entomologia* 41, 29-73.
