# Supplementary material for: The leaf beetle Labidostomis lusitanica (Coleoptera: Chrysomelidae) as an Iberian pistachio pest: projecting risky areas
Source: Pest Manag Sci. 2021 Sep 16;78(1):217–29. doi: 10.1002/ps.6624 (PMC9293163; doi:10.1002/ps.6624)
Supplement: Supplementary file 4 — Table S2. Data retrieved from the surveys sent to pistachio growers, with fields ordered by increasing total area. Attack probability is 0 or 1 if farmers report no or at least one observation of L. lusitanica on the trees. Estimated impact (only if answer was 1 for attack probability) was evaluated by the farmers choosing one of three tanks of perceived tree loss (0 = no loss, 1 = <10% loss, 2 = >10% loss). NA, not applicable; ‐, no answer was provided. [file PS-78-217-s001.docx]

**Table S2.** Data retrieved from the surveys sent to pistachio growers, with crop fields ordered by increasing total area. Attack probability is 0 or 1 if farmers report no or at least one observation of *L. lusitanica* on the trees. Estimated impact (only if answer was 1 for attack probability) was evaluated by the farmers choosing one of three ranks of perceived tree loss (0 = no loss, 1 = less than 10% loss, 2 = more than 10% loss). NA = not applicable. - indicates that no answer was provided.

| **Location** | **Province** | **Total field area (ha)** | **Attack probability by *L. lusitanica* (binary)** | **Estimated impact by *L. lusitanica* (loss rank)** | **% field area of 0-3 years (ha)** | **% field area of 4-8 years (ha)** | **% field area of >8 years (ha)** |
| --- | --- | --- | --- | --- | --- | --- | --- |
| Alcázar de San Juan | Ciudad Real | 0.70 | 1 | 0 | 0.00 | 0.00 | 100.00 |
| Alhama de Granada | Granada | 0.72 | 1 | 0 | 68.06 | 26.39 | 5.56 |
| Villarrobledo | Albacete | 0.80 | 1 | 1 | 0.00 | 100.00 | 0.00 |
| Cambil | Jaen | 0.80 | 1 | 2 | 0.00 | 100.00 | 0.00 |
| Almagro | Ciudad Real | 1.00 | 1 | 0 | 0.00 | 100.00 | 0.00 |
| Huéneja | Granada | 1.00 | 1 | 0 | 0.00 | 100.00 | 0.00 |
| Puebla de Guzmán | Huelva | 1.00 | 1 | 0 | 0.00 | 100.00 | 0.00 |
| Santa Cruz de los Cañamos | Ciudad Real | 1.00 | 1 | 0 | 100.00 | 0.00 | 0.00 |
| Begijar | Jaen | 1.00 | 1 | 2 | 100.00 | 0.00 | 0.00 |
| Toro | Zamora | 1.00 | 0 | NA | 0.00 | 100.00 | 0.00 |
| Montejicar | Granada | 1.00 | 1 | NA | - | - | - |
| San Miguel de la ribera | Zamora | 1.30 | 0 | NA | 0.00 | 0.00 | 100.00 |
| Villarrobledo | Albacete | 1.50 | 1 | 0 | 100.00 | 0.00 | 0.00 |
| Calabazas de Fuentidueña | Segovia | 1.50 | 0 | 0 | 100.00 | 0.00 | 0.00 |
| Alhama de Granada | Granada | 1.50 | 1 | 0 | - | - | - |
| Cuenca | Cuenca | 1.50 | 1 | 1 | 0.00 | 100.00 | 0.00 |
| Cuenca | Cuenca | 1.50 | 1 | 1 | 0.00 | 100.00 | 0.00 |
| Malagón | Ciudad Real | 1.50 | 1 | 1 | 0.00 | 0.00 | 100.00 |
| Cigales | Valladolid | 1.50 | 1 | 1 | 33.33 | 66.67 | 0.00 |
| Bailen | Jaen | 1.85 | 1 | 0 | 51.35 | 48.65 | 0.00 |
| Almagro | Ciudad Real | 2.00 | 1 | 0 | 0.00 | 100.00 | 0.00 |
| Escacena del Campo | Huelva | 2.00 | 1 | 0 | 0.00 | 100.00 | 0.00 |
| Alhambra | Ciudad Real | 2.00 | 1 | 0 | 0.00 | 0.00 | 100.00 |
| Madrigueras | Albacete | 2.00 | 1 | 0 | 100.00 | 0.00 | 0.00 |
| Algatocin | Málaga | 2.00 | 1 | 0 | - | - | - |
| Daimiel | Ciudad Real | 2.00 | 1 | 1 | 100.00 | 0.00 | 0.00 |
| Arahal | Sevilla | 2.00 | 1 | 1 | 100.00 | 0.00 | 0.00 |
| Torrenueva | Ciudad Real | 2.00 | 1 | 2 | - | - | - |
| Pozohondo | Albacete | 2.00 | 0 | NA | 0.00 | 100.00 | 0.00 |
| Colmenar de oreja | Madrid | 2.20 | 1 | 0 | 45.45 | 54.55 | 0.00 |
| Daimiel | Ciudad Real | 2.30 | 1 | 0 | 100.00 | 0.00 | 0.00 |
| Alhambra | Ciudad Real | 2.50 | 0 | 0 | 0.00 | 100.00 | 0.00 |
| Cambil | Jaen | 2.50 | 1 | 0 | 40.00 | 60.00 | 0.00 |
| Manzanares | Ciudad Real | 2.60 | 1 | 0 | 0.00 | 0.00 | 100.00 |
| Almodóvar del Campo | Ciudad Real | 2.75 | 0 | 0 | 100.00 | 0.00 | 0.00 |
| La Roda | Albacete | 3.00 | 1 | 0 | 0.00 | 100.00 | 0.00 |
| Socuéllamos | Ciudad Real | 3.00 | 1 | 0 | 0.00 | 100.00 | 0.00 |
| Alcazar de san juan | Ciudad Real | 3.00 | 1 | 0 | 0.00 | 100.00 | 0.00 |
| Benamaurel Granada | Granada | 3.00 | 1 | 0 | 33.33 | 66.67 | 0.00 |
| Hellin | Albacete | 3.00 | 0 | 0 | 100.00 | 0.00 | 0.00 |
| Albacete | Albacete | 3.00 | 1 | 0 | 100.00 | 0.00 | 0.00 |
| Daimiel | Ciudad Real | 3.00 | 1 | 0 | 100.00 | 0.00 | 0.00 |
| Cella | Teruel | 3.00 | 1 | 0 | 100.00 | 0.00 | 0.00 |
| Daimiel | Ciudad Real | 3.00 | 1 | 1 | 0.00 | 100.00 | 0.00 |
| Alcazar de San Juan | Ciudad Real | 3.00 | 1 | 2 | - | - | - |
| Pedro Martínez | Granada | 3.00 | 1 | NA | 66.67 | 33.33 | 0.00 |
| Valdepeñas | Ciudad Real | 3.00 | 0 | NA | - | - | - |
| Cabañas de Aliste | Zamora | 3.00 | 0 | NA | - | - | - |
| Cózar | Ciudad Real | 3.17 | 1 | 0 | 100.00 | 0.00 | 0.00 |
| Daimiel | Ciudad Real | 3.50 | 1 | 0 | 0.00 | 100.00 | 0.00 |
| Fuente el Fresno | Ciudad Real | 3.50 | 0 | 0 | 57.14 | 42.86 | 0.00 |
| El Bonillo | Albacete | 3.50 | 0 | 0 | 71.43 | 28.57 | 0.00 |
| Villarrobledo | Albacete | 3.50 | 1 | 0 | 100.00 | 0.00 | 0.00 |
| Villarrobledo | Albacete | 3.50 | 1 | 0 | - | - | - |
| Albatana | Albacete | 3.50 | 0 | NA | 100.00 | 0.00 | 0.00 |
| Albatana | Albacete | 3.50 | 0 | NA | 100.00 | 0.00 | 0.00 |
| Pozuelo de Calatrava | Ciudad Real | 3.87 | 1 | 0 | - | - | - |
| Fuente del Fresno | Ciudad Real | 4.00 | 1 | 0 | 0.00 | 100.00 | 0.00 |
| Calzada de Calatrava | Ciudad Real | 4.00 | 1 | 0 | 0.00 | 100.00 | 0.00 |
| Fernán Caballero | Ciudad Real | 4.00 | 1 | 0 | 50.00 | 50.00 | 0.00 |
| La solana | Ciudad Real | 4.00 | 1 | 0 | 100.00 | 0.00 | 0.00 |
| Villarta de San Juan | Ciudad Real | 4.00 | 1 | 2 | 25.00 | 75.00 | 0.00 |
| Mahora y Valdeganga | Albacete | 4.00 | 0 | NA | 0.00 | 100.00 | 0.00 |
| Fernan Caballero | Ciudad Real | 4.38 | 0 | NA | - | - | - |
| Fernan Caballero | Ciudad Real | 4.50 | 1 | 0 | 0.00 | 100.00 | 0.00 |
| Villanueva de los Infantes | Ciudad Real | 4.50 | 1 | 0 | 100.00 | 0.00 | 0.00 |
| Villanueva de los Infantes | Ciudad Real | 5.00 | 1 | 0 | 0.00 | 100.00 | 0.00 |
| Pozuelo de Calatrava | Ciudad Real | 5.00 | 1 | 0 | 0.00 | 100.00 | 0.00 |
| Sisante | Cuenca | 5.00 | 1 | 0 | 0.00 | 100.00 | 0.00 |
| Sabiote | Jaen | 5.00 | 1 | 0 | 0.00 | 100.00 | 0.00 |
| Bolaños | Ciudad Real | 5.00 | 1 | 0 | 0.00 | 0.00 | 100.00 |
| Ciudad Real | Ciudad Real | 5.00 | 1 | 0 | 0.00 | 0.00 | 100.00 |
| Almodovar del Campo | Ciudad Real | 5.00 | 1 | 0 | 100.00 | 0.00 | 0.00 |
| Montiel c Real | Ciudad Real | 5.00 | 1 | 0 | 100.00 | 0.00 | 0.00 |
| Santisteban del Puerto | Jaen | 5.00 | 1 | 0 | 100.00 | 0.00 | 0.00 |
| Tresjuncos | Cuenca | 5.00 | 1 | 0 | - | - | - |
| Villarobledo | Albacete | 5.00 | 1 | 1 | 0.00 | 100.00 | 0.00 |
| Villaconejos de Trabaque | Cuenca | 5.00 | 1 | 1 | 0.00 | 100.00 | 0.00 |
| Valdealgorfa | Teruel | 5.00 | 0 | NA | 0.00 | 100.00 | 0.00 |
| Manzanares | Ciudad Real | 5.50 | 1 | 0 | 0.00 | 100.00 | 0.00 |
| Huescar | Granada | 5.70 | 1 | NA | - | - | - |
| Cañaveras | Cuenca | 5.80 | 1 | 0 | 100.00 | 0.00 | 0.00 |
| Villarrobledo | Albacete | 6.00 | 1 | 0 | 0.00 | 100.00 | 0.00 |
| Membrilla | Ciudad Real | 6.00 | 1 | 0 | 0.00 | 100.00 | 0.00 |
| Villarrubia de los Ojos | Ciudad Real | 6.00 | 1 | 0 | 0.00 | 100.00 | 0.00 |
| Valdeganga | Albacete | 6.00 | 1 | 0 | 100.00 | 0.00 | 0.00 |
| Las Pedroñeras | Cuenca | 6.00 | 1 | 1 | 33.33 | 66.67 | 0.00 |
| Buciegas | Cuenca | 6.00 | 0 | NA | 100.00 | 0.00 | 0.00 |
| Elche de la Sierra | Albacete | 6.50 | 1 | 0 | 76.92 | 23.08 | 0.00 |
| Pozohondo | Albacete | 6.50 | 0 | NA | 0.00 | 100.00 | 0.00 |
| La Roda | Albacete | 6.80 | 1 | 0 | 100.00 | 0.00 | 0.00 |
| Alcolea de Calatrava | Ciudad Real | 7.00 | 1 | 0 | 0.00 | 100.00 | 0.00 |
| Carrion de Calatrava | Ciudad Real | 7.00 | 1 | 0 | 0.00 | 100.00 | 0.00 |
| Bollullos Par del Condado | Huelva | 7.00 | 1 | 0 | 100.00 | 0.00 | 0.00 |
| Valdealgorfa | Teruel | 7.00 | 1 | 0 | - | - | - |
| Villanueva de los Infantes | Ciudad Real | 8.00 | 1 | 0 | 0.00 | 100.00 | 0.00 |
| Los Pozuelos | Ciudad Real | 8.00 | 0 | 0 | 0.00 | 0.00 | 100.00 |
| Benamaurel | Granada | 8.00 | 1 | 0 | 50.00 | 50.00 | 0.00 |
| Vilches | Jaen | 8.00 | 1 | 0 | 50.00 | 50.00 | 0.00 |
| Membrilla | Ciudad Real | 8.00 | 1 | 0 | 100.00 | 0.00 | 0.00 |
| Pedrosa del Rey | Valladolid | 8.00 | 1 | 0 | 100.00 | 0.00 | 0.00 |
| Villamayor y Argamasilla de Calatrava | Ciudad Real | 8.00 | 1 | 1 | 100.00 | 0.00 | 0.00 |
| Tomelloso | Ciudad Real | 8.00 | 1 | 2 | 0.00 | 100.00 | 0.00 |
| Tomelloso | Ciudad Real | 8.00 | 1 | 2 | 0.00 | 100.00 | 0.00 |
| Tarancón | Cuenca | 8.00 | 1 | 2 | 33.75 | 66.25 | 0.00 |
| Tomelloso | Ciudad Real | 8.00 | 1 | 2 | 100.00 | 0.00 | 0.00 |
| Piedrabuena | Ciudad Real | 8.00 | 0 | NA | 0.00 | 100.00 | 0.00 |
| Piedrabuena | Ciudad Real | 8.00 | 0 | NA | 0.00 | 100.00 | 0.00 |
| Manzanares , Membrilla y Alhambra | Ciudad Real | 8.00 | 0 | NA | 81.25 | 18.75 | 0.00 |
| Manzanares , Membrilla y Alhambra | Ciudad Real | 8.00 | 0 | NA | 81.25 | 18.75 | 0.00 |
| Fuente Obejuna | Córdoba | 8.50 | 1 | 0 | 52.94 | 47.06 | 0.00 |
| Daimiel | Ciudad Real | 9.00 | 0 | 0 | 0.00 | 100.00 | 0.00 |
| Alamgro | Ciudad Real | 9.00 | 1 | 0 | 0.00 | 100.00 | 0.00 |
| Gobernador | Jaen | 9.00 | 1 | 0 | 100.00 | 0.00 | 0.00 |
| Villar de Cañas | Cuenca | 9.00 | 1 | 1 | - | - | - |
| Manzanares | Ciudad Real | 9.69 | 1 | 0 | 35.29 | 64.71 | 0.00 |
| Horcajo de las Torres | Ávila | 10.00 | 0 | 0 | 0.00 | 100.00 | 0.00 |
| Casas de Haro | Cuenca | 10.00 | 0 | 0 | 0.00 | 0.00 | 100.00 |
| Villamayor de Santiago | Cuenca | 10.00 | 1 | 0 | 100.00 | 0.00 | 0.00 |
| Jaen | Jaen | 10.00 | 1 | 0 | 100.00 | 0.00 | 0.00 |
| Piedrabuena | Ciudad Real | 10.00 | 0 | NA | 0.00 | 100.00 | 0.00 |
| Manzanares | Ciudad Real | 10.00 | 1 | NA | 0.00 | 30.00 | 70.00 |
| Casas de Juan Nuñez | Albacete | 10.00 | 0 | NA | 0.00 | 0.00 | 100.00 |
| La Solana, Membrilla, Manzanares | Ciudad Real | 10.50 | 1 | 0 | 52.38 | 0.00 | 47.62 |
| Daimiel | Ciudad Real | 11.00 | 1 | NA | 40.91 | 59.09 | 0.00 |
| Mollina | Málaga | 11.00 | 0 | NA | - | - | - |
| Daimiel | Ciudad Real | 11.50 | 1 | 0 | 39.13 | 60.87 | 0.00 |
| Alamedilla | Granada | 12.00 | 1 | 0 | 0.00 | 100.00 | 0.00 |
| Villarrobledo | Albacete | 12.00 | 1 | 0 | 0.00 | 50.00 | 50.00 |
| C. P. 23350 | Jaen | 12.00 | 1 | 0 | - | - | - |
| Cozar | Ciudad Real | 13.00 | 1 | 0 | 0.00 | 100.00 | 0.00 |
| Torreperogil | Jaen | 13.00 | 1 | 0 | 0.00 | 100.00 | 0.00 |
| Pozo Alcon | Jaen | 13.00 | 1 | 0 | 0.00 | 86.15 | 13.85 |
| Villahermosa | Ciudad Real | 13.00 | 0 | 0 | 100.00 | 0.00 | 0.00 |
| El Bonillo | Albacete | 13.00 | 0 | NA | 0.00 | 100.00 | 0.00 |
| Alhambra | Ciudad Real | 13.00 | 0 | NA | 38.46 | 38.46 | 23.08 |
| San Clemente | Cuenca | 14.00 | 1 | 0 | 0.00 | 0.00 | 100.00 |
| Alhambra | Ciudad Real | 14.00 | 0 | 0 | 42.86 | 0.00 | 57.14 |
| Socuellamos | Ciudad Real | 14.00 | 1 | 1 | 0.00 | 0.00 | 100.00 |
| Dólar | Granada | 15.00 | 1 | 0 | 80.00 | 20.00 | 0.00 |
| Viso del Marques | Ciudad Real | 15.00 | 0 | 0 | 100.00 | 0.00 | 0.00 |
| Villaescusa de Haro | Cuenca | 15.00 | 1 | 1 | 66.67 | 33.33 | 0.00 |
| Cózar | Ciudad Real | 18.00 | 1 | 0 | 0.00 | 0.00 | 100.00 |
| Almendros | Cuenca | 19.00 | 1 | 0 | 100.00 | 0.00 | 0.00 |
| Manzanares | Ciudad Real | 20.00 | 1 | 0 | 0.00 | 100.00 | 0.00 |
| Manzanares | Ciudad Real | 20.00 | 1 | 0 | 50.00 | 50.00 | 0.00 |
| Casas de Guijarro | Cuenca | 20.00 | 1 | 0 | 100.00 | 0.00 | 0.00 |
| Villanueva de los Infantes | Ciudad Real | 20.00 | 1 | 0 | - | - | - |
| Honrubia | Albacete | 20.00 | 1 | 1 | 100.00 | 0.00 | 0.00 |
| Villamayor de Santiago | Cuenca | 23.00 | 1 | 0 | 56.52 | 43.48 | 0.00 |
| Tobarra | Albacete | 23.00 | 0 | 0 | 86.96 | 13.04 | 0.00 |
| Manzanares | Ciudad Real | 25.50 | 1 | 1 | 14.90 | 29.41 | 55.69 |
| La Bóveda de Toro | Zamora | 26.50 | 1 | 0 | 71.70 | 28.30 | 0.00 |
| Villarrubia de los Ojos | Ciudad Real | 27.50 | 1 | 0 | 36.36 | 54.55 | 9.09 |
| Puebla de Almenara | Cuenca | 30.00 | 1 | 0 | 20.00 | 80.00 | 0.00 |
| Robledo | Albacete | 30.00 | 1 | 0 | 100.00 | 0.00 | 0.00 |
| Tomelloso | Ciudad Real | 32.33 | 1 | 0 | 22.58 | 44.23 | 33.19 |
| Villarobledo | Albacete | 40.50 | 1 | 0 | 33.33 | 54.32 | 12.35 |
| Villarta de San Juan | Ciudad Real | 44.00 | 0 | 0 | 45.45 | 22.73 | 31.82 |
| Albacete | Albacete | 54.00 | 0 | NA | 100.00 | 0.00 | 0.00 |
| Casasimarro | Cuenca | 56.00 | 0 | 0 | 100.00 | 0.00 | 0.00 |
| Malagon | Ciudad Real | 61.00 | 1 | 1 | 0.00 | 95.08 | 4.92 |
| Pozuelo de Calatrava | Ciudad Real | 100.00 | 1 | 0 | 0.00 | 0.00 | 100.00 |
| Villamalea | Albacete | 100.00 | 1 | 0 | 35.00 | 35.00 | 30.00 |
| Archidona | Málaga | 119.00 | 1 | 0 | 16.81 | 62.18 | 21.01 |
